# Aminopropyltriethoxysilane (APTES)-Modified Nanohydroxyapatite (nHAp) Incorporated with Iron Oxide (IO) Nanoparticles Promotes Early Osteogenesis, Reduces Inflammation and Inhibits Osteoclast Activity

**DOI:** 10.3390/ma15062095

**Published:** 2022-03-11

**Authors:** Krzysztof Marycz, Katarzyna Kornicka-Garbowska, Adrian Patej, Paulina Sobierajska, Andrzej Kotela, Eliza Turlej, Martyna Kepska, Alina Bienko, Rafal J. Wiglusz

**Affiliations:** 1 The Department of Experimental Biology, Faculty of Biology and Animal Science, Wroclaw University of Environmental and Life Sciences, Norwida 27B, 50-375 Wroclaw, Poland; katarzyna.kornicka-garbowska@upwr.edu.pl (K.K.-G.); eliza.turlej@upwr.edu.pl (E.T.); martyna.kepska@upwr.edu.pl (M.K.); 2 International Institute of Translational Medicine, Jesionowa 11, Malin, 55-114 Wisznia Mała, Poland; 3 Collegium Medicum, Cardinal Stefan Wyszynski University (UKSW), Woycickiego 1/3, 01-938 Warsaw, Poland; andrzejkotela@gmail.com; 4 Institute of Low Temperature and Structure Research, Polish Academy of Sciences, Okolna 2, 50-422 Wroclaw, Poland; a.patej@intibs.pl (A.P.); p.sobierajska@intibs.pl (P.S.); 5 Faculty of Chemistry, University of Wroclaw, F. Joliot-Curie14 Street, 50-383 Wroclaw, Poland; alina.bienko@uwr.edu.pl

**Keywords:** osteoporosis, osteoblasts, osteoclasts, hydroxyapatite, APTES, iron oxides

## Abstract

Due to its increased prevalence, osteoporosis (OP) represents a great challenge to health care systems and brings an economic burden. To overcome these issues, treatment plans that suit the need of patients should be developed. One of the approaches focuses on the fabrication of personalized biomaterials, which can restore the balance and homeostasis of disease-affected bone. In the presented study, we fabricated nanometer crystalline hydroxyapatite (nHAp) and iron oxide (IO) nanoparticles stabilized with APTES and investigated whether they can modulate bone cell metabolism and be useful in the fabrication of personalized materials for OP patients. Using a wide range of molecular techniques, we have shown that obtained nHAp@APTES promotes viability and RUNX-2 expression in osteoblasts, as well as reducing activity of critical proinflammatory cytokines while inhibiting osteoclast activity. Materials with APTES modified with nHAp incorporated with IO nanoparticles can be applied to support the healing of osteoporotic bone fractures as they enhance metabolic activity of osteoblasts and diminish osteoclasts’ metabolism and inflammation.

## 1. Introduction

Osteoporosis (OP) represents the common metabolic disease in human beings characterized by low bone mass and deteriorated microarchitecture, which results in reduced bone mechanical properties and finally bone fractures. For that reason, OP is a major health concern worldwide, especially in well-developed countries with aging populations and longer life spans. Although OP is more common in Caucasians, women and elderly patients, numerous people of both sexes and all races suffer from it, and the number of osteoporotic patients is rapidly growing [1]. As reported by the World Health Organization (WHO), one in three women and one in five men over the age of 50 will suffer a fracture caused by weak bones, while by 2050 the osteoporosis-related hip fracture incidence worldwide will increase by 240% in women, compared to data from the 1990s [2]. These alarming data highlight the need to develop novel, personalized materials for osteoporotic-related fracture bone regeneration.

Osteoporosis-related bone fractures are initiated by disturbed bone remodeling, the process in which the rate of bone resorption surpasses that of bone formation, leading in consequence to reduced bone mass [3]. In the physiological state, during the growth, bone formation exceeds bone resorption, resulting in bone expansion and increased bone mass and mechanical properties. The main goal of bone remodeling is to remove old bone and replace it with new bone. This in turn allows the regeneration or repair of small microfractures to prevent bone fractures, thereby assisting in maintaining a healthy skeleton. In elderly patients, as well as during menopause, the imbalance between resorption and formation rates leads to an increased risk of bone fractures. This process is mediated by two main bone cell populations: osteoblasts and osteoclasts [4]. The differentiated mesenchymal stem progenitor cells, i.e., osteoblasts that are involved in the synthesis of the bone matrix, including collagens, bone proteins and growth factors, lead to osteocyte maturation and formation of mineralized connective tissue. The process of osteoblast lineage cells’ maturation is modulated by several genes. The *Runx2* transcription factor (Cbfa1/AML3) plays a central role in the regulation of bone formation and modulates the expression of other genes involved in the process of osteoblast differentiation. *Runx2* exerts a wide spectrum of functions that are critical for bone formation, such as promoting the cell fate and controlling their growth pattern and proliferative activity [5]. *Runx2* is also involved in the regulation of gene activation and repression, support of chromatin remodeling and integration of extracellular matrix (ECM) signaling with hormonal transduction pathways. Once the osteoblasts have finished the synthesis of ECM, they become a cell that lines the bone surface and undergo cell death (apoptosis) or fusion with osteoclastic precursors, which results in formation of multinucleated osteoclasts that attach to the bone surface and commence resorption [6]. This process was shown to be mediated by a variety of cytokines, including interleukin IL-1, IL-6 and IL-7; tumor necrosis factor (TNFα); and granulocyte/macrophage colony-stimulating factor (GM-CSF). It has been demonstrated that under osteoporosis conditions, the higher expression of IL-1, IL-6 and TNFα in monocytes and/or osteoblasts and stromal cells occurs, which suggests that the bone inflammation becomes an integrative part of OP [7]. It is assumed that inflammation directly initiates the bone resorption process. By using a wide variety of enzymes, as well as hydrogen ions, osteoclasts break down the bone matrix, which initiates the bone resorption process. As the bone is composed of inorganic (hydroxyapatite) and organic fractions (collagen, proteoglycans and glycoproteins), the resorption process leaves the impaired microarchitecture of the bone matrix, which in turn activates an infiltration of macrophages to degrade and deposit organic material while releasing growth factors to initiate the bone deposition phase. The natural strategy, which targets reduction in osteoporotic-related bone fractures, includes the modulation of osteoblast activity while inhibiting osteoclast activity to prevent bone damage.

(3-Aminopropyl)triethoxysilane (APTES) is most often coupled via vapor-phase deposition, although other attempts have also resulted in sufficient coating quality. The reactions with nonpolar organic solvents also form highly stable polymer surface layers [8,9]. In aqueous solutions, a shell is generated through binding the silanol groups created during hydrolysis with hydroxyl groups on the hydroxyapatite surface via hydrogen bonds or electrostatic interactions [9,10], whereas amine groups can be utilized for further functionalization with organic biomolecules [11,12].

Nanohydroxyapatite (nHAp), with the chemical formula Ca_10_(PO_4_)_6_(OH)_2_, is the major inorganic component of the bone and teeth. Due to its well-known properties, such as structural similarity to natural bone, high porosity and excellent biocompatibility as well as osteoconductivity and osteoinductivity, nHAp is utilized as, implant coating, scaffolds and a drug delivery agent for tissue engineering and regenerative medicine [13,14,15,16]. However, nHAp possesses some imperfections, including fragility and high surface energy, causing difficulties in surface modification and leading nanoparticles to agglomerate, which decreases the mechanical properties of the material [8,17,18,19]. Hence, nHAp can be conjugated with polymeric layer covers to enhance its mechanical features, biocompatibility and enhance biological functionalization and thus improve its regenerative properties [13,20]. Moreover, nHAp scaffolds can also be combined with magnetic nanoparticles; magnetite (Fe_3_O_4_) is widely utilized for this purpose. Magnetite is known for its superparamagnetic properties, which allow the particles to be controlled by an external magnetic field with negligible remanence when turned off. The use of these nanoparticles as a drug delivery/release agent or contrast agents for magnetic resonance imaging (MRI) is necessary [21,22,23]. The conjugation of magnetite nanoparticles with nHAp can also improve the proliferation and growth of osteoblast cells and positively affect the osteoinductive properties of the obtained scaffolds, which allows the use of this combination in hyperthermia [24,25]. Nevertheless, it is necessary to cover magnetic particles because of their nanoscale toxicity to the human body [26]. Combining nHAp with Fe_3_O_4_ magnetic nanoparticles and a polymeric cover enables the maximization of composite biocompatibility used in a regenerative context.

In this study, we created nanometer crystalline hydroxyapatite (nHAp) and iron oxide (IO, Fe_3_O_4_) nanoparticles stabilized with APTES for novel orthopedic applications. Using a wide range of molecular techniques, we have shown that obtained nHAp@APTES promotes viability and *RUNX-2* expression in osteoblasts and reduces activity of critical proinflammatory cytokines while inhibiting osteoclast activity. The obtained data strongly support our hypothesis that nHAp@APTES might become a novel material for osteoporotic-related bone fracture regeneration.

## 2. Materials and Methods

### 2.1. Synthesis of Iron Oxide Nanoparticles (IO)

Fabrication of the magnetic nanoparticles was carried out by using wet chemical coprecipitation according to our previous paper [27]. The precursors were as follows: FeSO_4_ ∙ 7H_2_O (Chempur, Piekary Slaskie, Poland, <99.5%), KOH (Chempur, Piekary Slaskie, Poland, pure p.a.) and KNO_3_ (Chempur, Piekary Slaskie, Poland, <99%). At the beginning, hot water solutions of 3.5712 g (12.85 mmol) of FeSO_4_∙7H_2_O, 0.4329 g (4.28 mmol) of KOH and 1.4415 g (25.69 mmol) of KNO_3_ were prepared. Subsequently, to dissolve FeSO_4_∙7H_2_O, the KNO_3_ and KOH solutions were added dropwise, respectively, and stirred at 90 °C for 10 min afterwards. The obtained black precipitate was separated from the by-products with a magnet then washed with deionized water to obtain neutral pH and further dried at 70 °C for 24 h.

### 2.2. Synthesis of Hydroxyapatite Nanoparticles (nHAp)

To obtain the nHAp nanoparticles, microwave-stimulated hydrothermal technique based on the preparation methods described by P. Sobierajska and R.J. Wiglusz was applied [28]. The following reagents were used: Ca(NO_3_)_2_·4H_2_O (Sigma-Aldrich, St. Louis, MO, USA, >99%), (NH_4_)_2_HPO_4_, (Acros Organics, Geel, Belgium, 98+%) and C_6_H_8_O_7_ (Alfa Aesar, Haverhill, MA, USA, 99+%). In the first step, 1.0577 g (4.48 mmol) of calcium nitrate tetrahydrate and 0.1721 g (0.08 mmol) of citric acid were dissolved in 25 mL of deionized water. Subsequently, water solution of 1.0647 g (8.06 mmol, 3-fold excess relative to the calcium ion concentration) diammonium hydrogen phosphate and 5 mL of ammonia (25%) were added, respectively. The obtained mixture was transferred to the Teflon vessel and put in a microwave reactor (Magnum II, ERTEC-Poland, Wrocław, Poland) under the pressure of 20–30 atm at 200 °C for 90 min. Afterwards, the obtained powder was washed with deionized water to obtain neutral pH and further dried in 70 °C for 24 h.

### 2.3. Synthesis of Nanohydroxyapatite–Iron Oxide Composite (nHAp/IO)

Altered microwave-stimulated hydrothermal method proposed by D. Karthickraja et al. [29] was applied to obtain the nHAp/IO composite. Initially, 20 mL of a water suspension of 0.3000 g (1.3 mmol) of iron oxide (IO) and 1.0647 g (8.06 mmol, 3-fold excess relative to the calcium ion concentration) of diammonium hydrogen phosphate ((NH_4_)_2_HPO_4_, Acros Organics, Geel, Belgium, 98+%) was sonicated for 15 min. Afterwards, 5 mL of ammonia (25%) was added to the mixture to obtain alkaline environment. Then, 25 mL of water solution of 1.0577 g (4.48 mmol) of calcium nitrate (Ca(NO_3_)_2_, Sigma-Aldrich, St. Louis, MO, USA, > 99%) and 0.1721 g (0.08 mmol) of citric acid (C_6_H_8_O_7_, Alfa Aesar, Haverhill, MA, USA, 99+%) were added, and the mixture was sonicated for 10 min. Subsequently, the obtained suspension was transferred to the Teflon vessel and put into a microwave reactor (Magnum II, ERTEC-Poland, Wrocław, Poland) under the pressure of 20–30 atm at 200 °C for 90 min. Finally, the synthesized product was washed with deionized water to remove the by-products and further dried at 70 °C for 24 h.

### 2.4. Surface Modification of nHAp, nHAp/IO and IO Using APTES

The APTES coverage was carried out using the method described by C. S. Goonasekera et al. [30]. A total of 0.2 g of the obtained nHAp or nHAp/IO was added to 20 mL of 1% APTES (Alfa Aesar, Haverhill, MA, USA, 98%) solution in n-hexane (POCH, Gliwice, Poland, pure p.a.) and further stirred for 3 h. The white (nHAp@APTES) and brown precipitate (IO@APTES) were separated, respectively, from the solution using a centrifuge (10,000 rpm, 5 min), washed a few times in n-hexane and dried at 70 °C for 24 h. Afterwards, material was heat-treated at 150 °C for 24 h.

A total of 0.4 g of the obtained IO was added to 10 mL of 1% APTES solution (Alfa Aesar, Haverhill, MA, USA, 98%) in n-hexane (POCH, Gliwice, Poland, pure p.a.) and further stirred for 24 h. The black precipitate (IO@APTES) was magnetically separated from the solution, washed in n-hexane and dried at 70 °C for 24 h.

### 2.5. Characterization of Fabricated nHAp@APTES, IO@APTES and nHAp/IO@APTES

To confirm purity of obtained materials, X-ray powder diffraction (XRPD) patterns were recorded in a 2θ range of 5–100 with X’Pert Pro PANalytical X-ray diffractometer (Cu Kα1: 1.54060 Å). Covering the materials with a polymer layer was verified by mid-IR (4000–400 cm^−1^ with 4 cm^−1^ spectral resolution) spectrum collected in KBr pellets using the Nicolet iS50 Fourier Transform Infrared Spectroscopy (FT-IR Thermo Scientific) spectrometer equipped with Automated Beamsplitter exchange system (iS50 ABX containing DLaTGS KBr detector), Thermo Scientific Polaris^TM^ and HeNe laser as an IR radiation source. The morphology and elemental analyses were carried out using the scanning electron microscope FEI Nova NanoSEM 230 equipped with an EDS spectrometer (EDAX PegasusXM4) and operating at an acceleration voltage in the range of 3.0–15 kV and spot 2.5–3.0. High-resolution transmission electron microscopy (HRTEM) images and selected area electron diffraction (SAED) patterns were recorded using a Philips CM-20 SuperTwin microscope operating at 160 kV. The TEM images were used to determine the average particle size through direct measurements of 100 particles. The measurements were performed using the ImageJ 1.53k software (National Institutes of Health, Rockville Pike, MD, USA). The obtained values were further presented in the histograms with kernel smoothing distribution.

Variable-temperature (1.8–300 K) direct-current (DC) magnetic susceptibility measurements under applied field of B = 0.01 T and variable-field (0–5 T) magnetization measurements at temperatures of 2 and 300 K were carried out with SQUID MPMS magnetometer (Quantum Design). The magnetic susceptibility data were corrected for the diamagnetism of the constituent atoms and the sample holder. DC measurements were carried out by crushing the crystals and restraining the sample in order to prevent any displacement due to its magnetic anisotropy.

### 2.6. Cell Lines

The murine osteoblast precursor cell line (MC3T3-E1) and murine monocyte–macrophage cell line (RAW 264.7) were obtained from EACC (European Collection of Authenticated Cell Cultures, Sigma-Aldrich, Munich, Germany). The preosteoclastic murine cell line (4B12) was a kind gift from Shigeru Amano from the Department of Oral Biology and Tissue Engineering, Meikai University School of Dentistry, in Japan. All of them were maintained in the Department of Experimental Biology, Wrocław University of Environmental and Life Science.

MC3T3-E1 cell line was cultured in MEMα without the addition of ascorbic acid (Minimum Essential Media Alpha, Gibco, Scotland, UK) supplemented with 10% FBS (Fetal Bovine Serum, Sigma-Aldrich, Munich, Germany). MC3T3-E1 cells were used to determine osteoinductive properties of investigated materials.

4B12 cell line was also cultured in MEMα supplemented with 10% FBS and additionally with 30% of CSCM medium (Calvaria-derived stromal cell-conditioned medium, DBE, Wrocław). 4B12 cell line was used to reveal whether fabricated materials exert antiosteoclastogenic properties.

In turn, RAW 264.7 cell line was cultured in DMEM medium (Dulbecco’s Modified Eagle Medium, with 10% FBS and with 4500 mg/L glucose (Sigma-Aldrich, Munich, Germany). RAW 264.7 macrophages were used to evaluate whether fabricated materials are immunogenic and if they modulate M1/M2 macrophage polarization.

All cell cultures were maintained in CO_2_ incubator at standard conditions (37 °C, 5% CO_2_, 95% humidity).

### 2.7. In Vitro Toxicology Assay

The kinetics of the impact of the new synthesized biomaterials nHAp, nHAp@APTES and IO@APTES and finally nHAp/IO@APTES on the cell viability of MC3T3-E1 and 4B12 cell line was assessed using in vitro toxicology assay kit based on the resazurin (Sigma-Aldrich, St. Louis, MO, USA) according to the manufacturer’s protocol. Briefly, the MC3T3-E1 cell line was seeded at the density of 1.25 × 10^4^/mL (2.5 × 10^3^ cells/well) or at the density of 1.75 × 10^4^/mL (3.5 × 10^3^ cells/well) in the case of 4B12 cell line on 96-well plastic plates and incubated in the presence of nHAp alone or with combinations of nHAp@APTES, IO@APTES and finally nHAp/IO@APTES in the concentration of 18.16 μg/mL for 20 h, 44 h and 72 h. Next, the rezaurin dye solution, in an amount of 10% of the culture medium volume, was added, and after 2 h, the absorption of the samples was measured using Elisa reader (Epoch, BioTek Instruments, Vinooski, VT, USA) equipped with Gen5 software (No 5). The results were obtained at the 560 nm wavelength. The presented results represent three independent experiments.

### 2.8. Mitochondria Network Evaluation

The evaluation of the impact of the new biomaterials on the mitochondria network was carried out using commercially available kit Mito Red (Sigma-Aldrich, St. Louis, MO, USA) according to the manufacturer’s protocol. Briefly, the MC3T3-E1 cell line was seeded on the plastic plates at the density of 1.4 × 10^4^/mL (7 × 10^3^ cells/well) or at the density of 2 × 10^4^/mL (10 × 10^3^ cells/well) in the case of the 4B12 cell line on 24-well plastic plates and were incubated in the presence of the biomaterials for 20 h. Then, the cells were treated with solution of 20 mM Mito Red in DMSO and incubated at 37 °C for 30 min. Additionally, F-actin antibody (Santa Cruz Biotechnology, Dallas, TX, USA) in concentration of 1:800 was added to each probe for 30 min. Slides were mounted with the Mounting Medium with DAPI (4,6-diamino-2-phenylindole, Thermo Fisher Scientific, Waltham, MA, USA). The slides were analyzed using Leica TCS SP8 confocal microscope (Leica Microsystems, Weltzar, Germany) equipped with Leica-LasX software (V4). The photographs were captured under 630× magnification and were analyzed using Fiji New ImageJ with the Colour Pixel Counter plugin version 1.52 developed by W. Rasband (NIH, Bethesda, MD, USA). Each experiment was performed in triplicate.

### 2.9. Western Blot

Protein amount in each sample was estimated with Pierce™ BCA Protein Assay Kit (Thermo Fisher Scientific, Waltham, MA, USA). Samples were subjected to SDS-polyacrylamide gel electrophoresis at 100 V for 90 min using Mini-PROTEAN Tetra Vertical Electrophoresis Cell (Bio-Rad, USA). Protein was transferred onto polyvinylidene difluoride (PVDF) membranes (Bio-Rad, Hercules, CA, USA) using a Mini Trans-Blot^®^ Cell (Bio-Rad, Hercules, CA, USA) at 100 V for 1 h at 4 °C. Blocking was performed by incubation of membranes in 5% nonfat milk in TBST for 2 h. Membranes were incubated with secondary HRP-conjugated antibodies (dilution 1:5000 in TBST for 2 h). Antibodies and their dilutions are shown in Table 1. Chemiluminescent signals were detected using Chemiluminescent/Fluorescent Substrate Kit (Vector Laboratories, Inc., Burlingame, CA, USA, SK-6604) with ChemiDoc MP Imaging System (Bio-Rad, Hercules, CA, USA) and quantified with Image Lab SoftwareV06 (Bio-Rad, Hercules, CA, USA).

### 2.10. Gene Expression Evaluation

The assessment of the gene expression was performed using RT-qPCR method. The MC3T3-E1 cell line was seeded at the density of 6 × 10^4^/mL (30 × 10^3^ cells/well) or at the density of 8 × 10^4^/mL (40 × 10^3^ cells/well) in the case of the 4B12 cell line on the 24-well plastic plates and were incubated with the biomaterials in the concentration of 18 μg/mL for 20 h. RNA was isolated from cells using Extrazol (Blirt DNA, Gdańsk, Poland). The quantity and the purity of RNA was measured using spectrophotometer at 260/280 nm wavelength (Epoch, BioTek Instruments, Winooski, VT, USA). In order to digest the gDNA and synthesize cDNA from 800 ng of RNA, Precision DNAse Kit (Primerdesign, Blirt DNA, Gdańsk, Poland) and Tetro cDNA Synthesis Kit (Bioline Reagents Limited, London, UK) were used according to the manufacturers’ protocol. The reverse transcription was performed using thermocycler T100 (BioRad, Hercules, CA, USA). The real-time qPCR was performed using SensiFAST SYBR Green & Fluorescein Kit (BioLine Reagents Ltd., London UK) and probes for *p21*, *p53*,*Casp 9*, *Bad*, *Bax* and *Bcl2* (apoptosis panel); *Mff*, *Mfn1* and *Pink1* (mitochondrial panel), *Runx2*, *Alp*, *Col1a-1*, *Opn*, *Bglap2*, *Dmp1*, *Mmp9*, *Itgav*, *PU.1* and *c-fos* (osteogenic/osteoclastogenic differentiation panel) and *IL1b*, *IL6*, *Tgfb1*, *Nos2* and *Tnf* (inflammation panel) using CFX Connect Real-Time PCR Detection System (BioRad, Hercules, CA, USA) equipped with BioRad CFX Maestro 1.0 software version 4.0.2325 0418. The analysis was performed according to the *GAPDH* house-keeping gene (Glceraldehyde 3-phosphatehydrogenase). Each experiment was performed independently three times. Primers are listed in Table 2.

### 2.11. Osteoblasts and Osteoclast Coculturing

The RAW 264.7 cell line was cocultured with MC3T3-E1 or 4B12 cell line to evaluate the stage of inflammation in relation to the new biomaterials. Briefly, RAW264.7 cell line was seeded on the 24-well plastic plates (Greiner Bio-One Suns Co., Ltd., Kremsmunster, Austria) at the density of 2 × 10^5^/well and maintained in the presence of LPS (lipopolysaccharide from *Pseudomonas aeruginosa*, Sigma-Aldrich, Munich, Germany) in the appropriate complete medium for 6 h followed by 20 h of incubation in the presence of new biomaterials in the concentration of 1 μg/mL. Next, the MC3T3-E1 cell line at the density of 8 × 10^3^/well or in the case of the 4B12 cell line at the density of 1 × 10^4^/well was added to the culture and coculture with RAW 264.7 cell line for 24 h.

### 2.12. Statistical Analysis

Obtained results are presented as the mean with standard deviation (±SD), derived from at least three technical repetitions. Statistical comparison was performed using t-Student test or one-way analysis of variance with Dunnett’s post hoc test. The calculation was performed using GraphPad Software (Prism 9.00, San Diego, CA, USA). Differences with a probability of *p* < 0.05 were considered as statistically significant.

## 3. Results

### 3.1. Structure of the Obtained Materials

**Figure 1:**
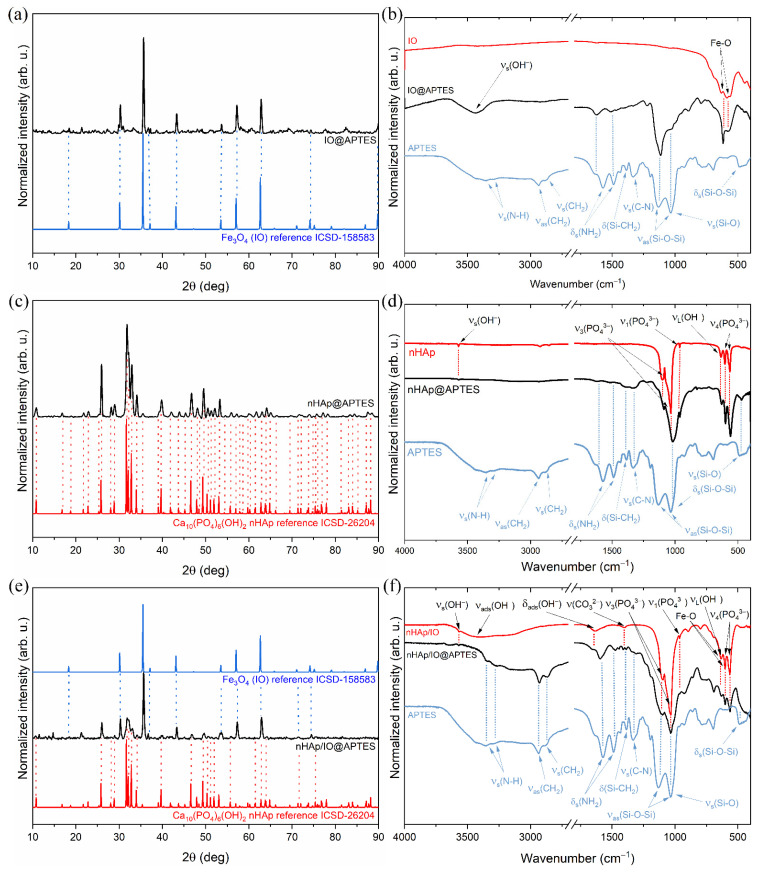
XRD patterns (left panel) and FT-IR spectra (right panel) of pure IO (**a**,**b**) and IO modified with APTES, pure nHAp and nHAp modified with APTES (**c**,**d**) as well as nHAp/IO and composite modified with APTES (**e**,**f**). Pure APTES was marked as blue line.

To investigate the structures of obtained materials, an X-ray diffractogram was recorded and compared with the Inorganic Crystal Structure Database standards (ICSD-26204 for hydroxyapatite [30] and ICSD-158583 for magnetite [31]). In the case of nHAp/IO@APTES (Figure 1e), the peaks at 2θ equal to 10.8°, 25.8°, 28.8°, 35.3°, 39.6°, 55.6°, 69.4°, 71.3°, 73.7°, 75.8°, 76.2° and 77.9° in the ranges from 31.6° to 33.9°, from 46.5° to 51.9° and from 61.5° to 64.8° are characteristic of the hexagonal hydroxyapatite structure (*P6_3_/m* spacer group, theoretical cell parameters: a = b = 9.424(4) Å, c = 6.879(4) Å and α = β = 90° γ = 120°). In turn, the peaks at 2θ equal to 30.1°, 35.5°, 37.1°, 43.1°, 53.4°, 57.0°, 62.6° and 74.1° have been assigned to the orthorhombic Fe_3_O_4_ cubic inverse spinel structure (*Fd3^−^m* space group, theoretical cell parameters: a = b = c = 8.394(1) Å and α = β = γ = 90° [31]). All detected diffraction peaks for nHAp@APTES, as shown in Figure 1c, overlapped with those belonging to the ICSD reference structure of the hexagonal hydroxyapatite. In the case of IO@APTES, as shown in Figure 1a, the peaks at 2θ equal to 18.3°, 30.1°, 35.6°, 36.7°, 43.2°, 53.6°, 57.2°, 62.8°, 74.2° and 89.9° have been ascribed to the orthorhombic Fe_3_O_4_.

The FT-IR spectrum of IO@APTES is shown in Figure 1b. The spectrum (marked as a red line) contained the vibration bands of iron oxide (IO, black line) belonging to the stretching vibration (*v_s_*(Fe-O)) of the Fe-O bond located at 582 and 635 cm^−1^. The stretching vibration (*v_s_*(OH^−^)) of adsorbed water molecules on the ferrite particle surface was located at 3427 cm^−1^ [32,33,34]. The bands that confirm the presence of APTES (blue line) in IO@APTES correspond to the positions at 1112, 1513 and 2930 cm^−1^ ascribed to asymmetric modes (ν_as_(Si-O-Si)) of Si-O-Si siloxane groups, deformation modes (δ_s_(NH_2_)) of the NH_2_ group and the symmetric vibration of the aminopropyl chain (ν_s_(CH_2_)), respectively [8,35,36,37]. For the amine group, symmetric stretching modes (*v_s_*(N-H)) were also matched at 3349 and 3282 cm^−1^ [8,36,37].

The FT-IR spectrum of obtained nHAp@APTES is presented in Figure 1d. The spectrum (marked as a black line) exhibited several bands characteristic of the (PO_4_^3−^) group of nHAp (red line), including the triply degenerate *v*_4_ bending vibration of O–P–O located at 565 and 602 cm^−1^ [38] as well as the symmetric nondegenerate *ν*_1_ stretching vibration occurring at 963 cm^−1^ and the antisymmetric triply degenerate *v*_3_ stretching vibration at 1031 and 1092 cm^−1^ [38,39,40]. Other bands were located at 632 and 3575 cm^−1^ and correspond to the librational mode (*v_L_*(OH^−^)) and the stretching vibration (*v_s_*(OH^−^)) of the hydroxyl group, respectively [38]. The APTES (blue line) polymer layer in the studied material was confirmed by the presence of bands assigned to asymmetric modes (ν_as_(Si-O-Si)) and bending modes (δ_s_(Si-O-Si)) of Si-O-Si siloxane groups as well as stretching vibrations (ν_s_(Si-O)) of the Si-O bond at 1009 and 470 cm^−1^, respectively [8,35,36,37]. Other characteristic peaks for APTES were located at 1337 and 1390 cm^−1^ and belong to stretching vibrations (ν_s_(C-N)) of C-N bond and deformation mode (δ_s_(Si-CH_2_)) of the Si-CH_2_ vibration [8,37,38], respectively. The peaks at 1629 and 1525 cm^−1^ correspond to the deformation modes (δ_s_(NH_2_)) of NH_2_ groups [8,35,36,37].

The FT-IR spectra for composite, APTES and composite covered with polymer are shown in Figure 1f. The spectrum of nHAp/IO@APTES (black line) exhibited typical bands for the pure composite (red line) including stretching vibrations (*v_s_*(Fe-O)) of the Fe-O bond from IO located at 580 and 634 cm^−1^ [32,33,34]. They overlapped with the librational mode (*v_L_*(OH^−^)) of hydroxyl group at 632 cm^−1^ as well as triply degenerate v_4_ bending vibration of O–P–O bonds of the PO_4_^3−^ group belonging to nHAp at 565 and 603 cm^−1^ [39]. Another phosphate group vibration is the symmetric nondegenerate *ν*_1_ stretching vibration located at 960 cm^−1^ and the antisymmetric triply degenerate *v*_3_ stretching vibration at 1031 and 1092 cm^−1^ [38,39,40]. The band at 1404 cm^−1^ is typical of C-O vibration of adsorbed CO_2_ or the CO_3_^2−^ group replacing PO_4_^3−^ [41,42,43,44] in the hydroxyapatite structure. The stretching vibration (*v_s_*(OH^−^)) of the hydroxyl group (3572 cm^−1^) is characteristic of nHAp, while the bending (δ_ads_(OH^−^)) mode at 1637 cm^−1^ and broad stretching vibration (δ_ads_(OH^−^)) at 3800–2800 cm^−1^ were derived from adsorbed water molecules on the material surface [28,33,40,43,45,46]. The presence of the polymer in nHAp/IO@APTES was confirmed by the presence of absorption peaks characteristic of pure APTES (blue line) located at 488, 1037 and 1135 cm^−1^ belonging to the scissoring mode (δ_s_(Si-O-Si)) and asymmetric modes (ν_as_(Si-O-Si)) of Si-O-Si siloxane groups, respectively. The peak at 1035 cm^−1^ indicates the stretching vibration (ν_s_(Si-O)) of the Si-O silanol group, whereas the band at 1435 cm^−1^ corresponds to the deformation mode (δ_s_(Si-CH_2_)) of the Si-CH_2_ vibration [8,36,37]. Another typical vibration for APTES is that ascribed to the stretching vibration (ν_s_(C-N)) of the C-N bond at 1332 cm^−1^ as well as deformation modes (δ_s_(NH_2_)) of NH_2_ at 1573 and 1489 cm^−1^. There was a symmetric nondegenerate *ν*_1_ stretching vibration located at 960 cm^−1^ and an antisymmetric triply degenerate *v*_3_ stretching vibration at 1031 and 1092 cm^−1^ [38,39,40]. The band at 1404 cm^−1^ is typical of the C-O vibration of adsorbed CO_2_ or CO_3_^2−^ groups replacing PO_4_^3−^ [41,42,43,44] in the hydroxyapatite structure. The stretching vibration (*v_s_*(OH^−^)) of the hydroxyl group (3572 cm^−1^) is characteristic of nHAp, while the bending (δ_ads_(OH^−^)) mode at 1637 cm^−1^ and broad stretching vibration (δ_ads_(OH^−^)) at 3800–2800 cm^−1^ were derived from adsorbed water molecules on the material surface [28,33,40,43,45,46]. The presence of the polymer in nHAp/IO@APTES was confirmed by the presence of absorption peaks characteristic of pure APTES (blue line) located at 488, 1037 and 1135 cm^−1^ belonging to the scissoring mode (δ_s_(Si-O-Si)) and asymmetric modes (ν_as_(Si-O-Si)) of Si-O-Si siloxane groups, respectively. The peak at 1035 cm^−1^ indicates the stretching vibration (ν_s_(Si-O)) of the Si-O silanol group, whereas the band at 1435 cm^−1^ corresponds to the deformation mode (δ_s_(Si-CH_2_)) of the Si-CH_2_ vibration [8,36,37]. Other typical vibrations of APTES are those ascribed to the stretching vibration (ν_s_(C-N)) of the C-N bond at 1332 cm^−1^ as well as deformation modes (δ_s_(NH_2_)) of NH_2_ at 1573 and 1489 cm^−1^. There are also asymmetric (ν_as_(N-H)) and symmetric stretching modes (ν_s_(N-H)) with maximum positions at 3349 and 3291 cm^−1^ [8,35,36,37]. Finally, the bands at 2886 and 2936 cm^−1^ can be ascribed to the asymmetric (ν_as_(CH_2_)) and symmetric (ν_s_(CH_2_)) vibration of the aminopropyl chain [8,36].

### 3.2. Quantitative Analysis, Morphology and Particle Size of the Obtained Materials

**Figure 2:**
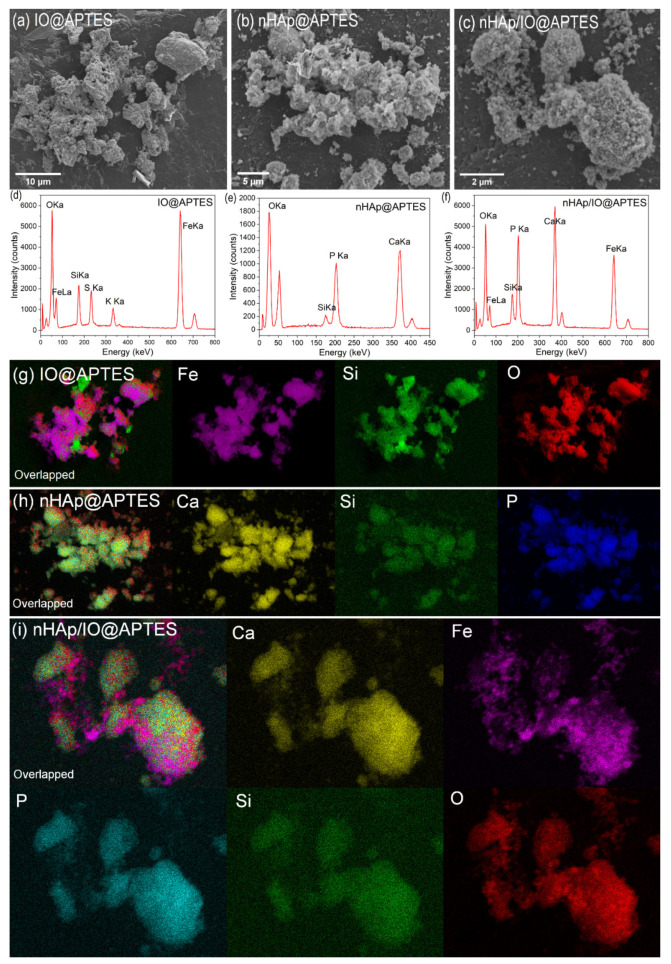
SEM images (**a**–**c**) and EDS analysis (**d**–**f**), as well as SEM-EDS elemental maps (**g**–**i**) of IO@APTES, nHAp@APTES and nHAp/IO@APTES, respectively.

To verify the success of covering all the studied materials with APTES, scanning electron microscopy together with energy disperse X-ray spectroscopy (SEM-EDS) was applied. Figure 2a,d present the SEM image and EDS spectrum of IO@APTES. The obtained material had a tendency to agglomerate. An effective coverage of the iron oxide by the polymer layer was confirmed by overlapped elemental maps of Fe, O and Si (Figure 2g). In the case of the EDS spectrum, the presence of K and S was detected, indicating there were residues after synthesis.

SEM images of nHAp@APTES presented in Figure 2b show the uniform but highly agglomerated surface of the material, which makes the single particles harder to distinguish. nHAp coverage by the APTES polymeric layer is visible due to the presence of the Si element for the same areas as for Ca and P elements belonging to nHAp (Figure 2h). The quantitative composition of the obtained nHAp@APTES was confirmed by EDS analysis, as shown in Figure 2e. The estimated Ca/P molar ratio equals 1.53, which is smaller than the theoretical value (1.67); nevertheless, it is still suitable due to the high structural stability of nHAp [47]. Figure 2c shows SEM image of nHAp/IO@APTES. The surface of the composite was homogenous, but it was hard to distinguish the single particles. The EDS analysis presented in Figure 2f confirmed the composition of the material. The elemental maps (see Figure 2i) showed that Fe and Ca occur in common areas and have a tendency towards mutual agglomeration, whereas the presence of Si in the same area as the other elements indicated the success of covering the composite with APTES.

**Figure 3:**
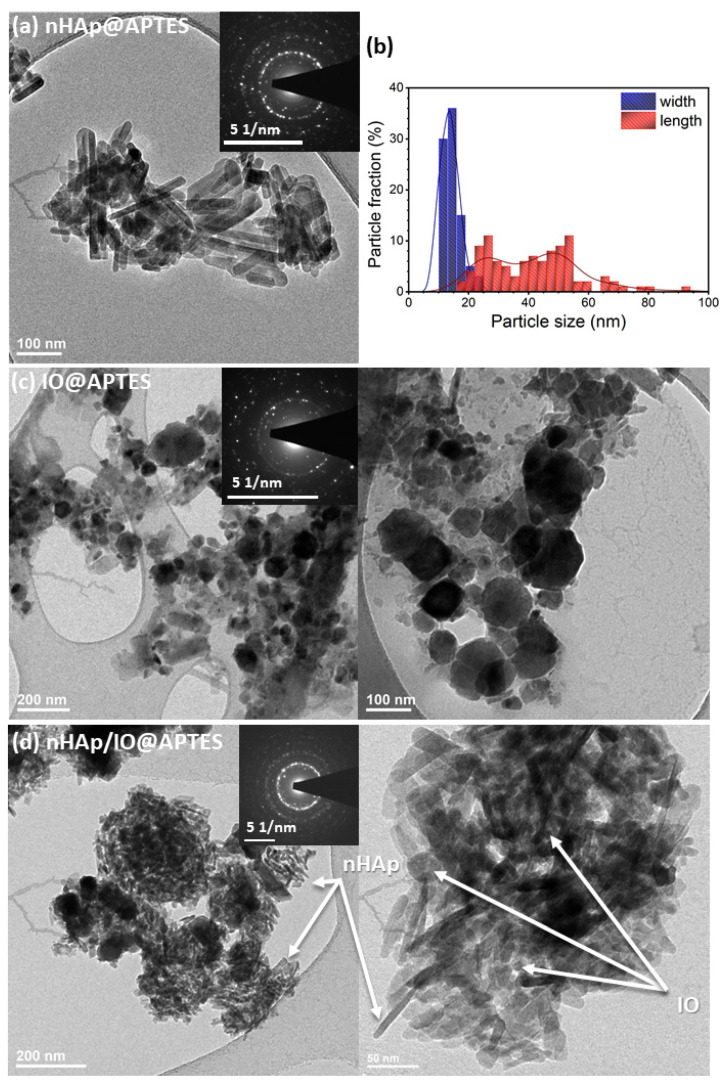
TEM and SAED (inset) images (**a**,**c**,**d**) of nHAp@APTES, IO@APTES and nHAp/IO@APTES, and the histogram (**b**) indicates the distribution of particles size of nHAp@APTES.

To examine the morphology and particle size of obtained materials, transmission electron microscopy images were collected. As can be seen in Figure 3a, nHAp@APTES particles were highly agglomerated and possessed an elongated shape in the form of nanorods with 2, 27 and 48 nm lengths and an average width of 14 nm, according to the size distribution histogram shown in Figure 3b. The selected area electron diffraction (SAED) image shows well-developed spotty rings indicating the nanopolycrystallinity of the studied sample (Figure 3a inset).

TEM images of IO@APTES (Figure 3c) show that iron oxide particles possessed a slightly similar sphere shape with an average diameter within the range of 30–80 nm and had a tendency towards agglomeration. SAED images (Figure 3c, inset) indicate that the obtained IO@APTES belongs to the polycrystalline material.

TEM images of the nHAp/IO@APTES are presented in Figure 3d. As can be seen, the elongated nHAp particles and spherical-like IO mutually agglomerated. Both components clearly interacted with each other. The most probable explanation for this is the occurrence of physical interactions since the results of measurements, especially FT-IR spectra, do not indicate the formation of new bonds. Additionally, the SAED image indicates the nanopolycrystallinity of the nHAp/IO@APTES due to the presence of well-developed spotty rings (Figure 3d, inset).

### 3.3. Magnetic Properties of nHAp/IO@APTES

**Figure 4:**
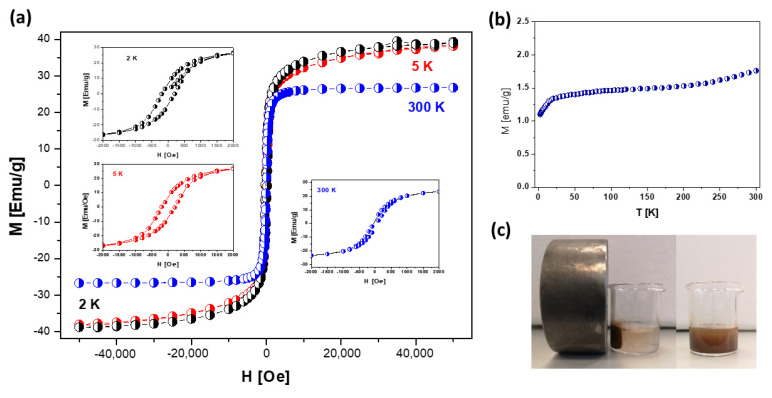
Field H dependence on magnetization M of nHAp/IO@APTES composite at 2 K (black circle), 5 K (red circle) and 300 K (blue circle) (**a**), temperature T dependence on magnetization M at 100 Oe (**b**) and composite dispersed in water in the presence and lack of external magnetic field (**c**).

The presence of magnetite nanoparticles upon examining the composite material was confirmed by magnetic measurements using a superconducting quantum interference device (SQUID) within the temperature range of 1.8–300 K (Figure 4b) with a magnetic field of 0–5T (Figure 4a).

The results clearly show the superparamagnetic behavior of nanoparticles. The magnetization in comparison to the magnetic field curve indicates a nonlinear variation at the measuring temperatures, 2, 5 and 300 K. The nHAp/IO@APTES was saturated at a low magnetic field with the Ms saturation magnetization value (obtained as an extrapolation to the zero field from the high-field area in M (H)) of 39.1 (at 2 K), 38.2 (at 5 K) and 26 EMU/g (at 300 K), respectively. The former was significantly lower than the saturation magnetization of bulk magnetite (Ms bulk = 98 EMU/g) [45], which may be the result of the final particle size effect and high surface-area-to-volume ratio, the spin deflection effect at the grain boundary or the presence of other materials in the composite structure that may lead to a reduction in the efficacy of the magnetic moment [46,48]. Nevertheless, the observation shown in Figure 4c still indicates sufficiently strong magnetic separation of the studied composite when a magnetic field was applied.

At 300 K, the coercivity (26.3 Oe) and remanence values (3.2 EMU/g) were not discernible, indicating a superparamagnetic behavior, while at 2 K, the value of coercivity (151 Oe for AP2) and remanence (6.9 EMU/g) showed a ferrimagnetic behavior. Additionally, the zero-field cooling curves were almost flat in the temperature range of 25–150 K and displayed a hump at T = 25 K. Above 150 K, this curve constantly increased, representing a rather ferrimagnetic property. The sharp fall in ZFC magnetization at Tf is indicative of the typical cooperative freezing (spin-glass-like) behavior of strongly interacting particles in a frustrated magnetic system [49,50].

### 3.4. Cytotoxicity

**Figure 5:**
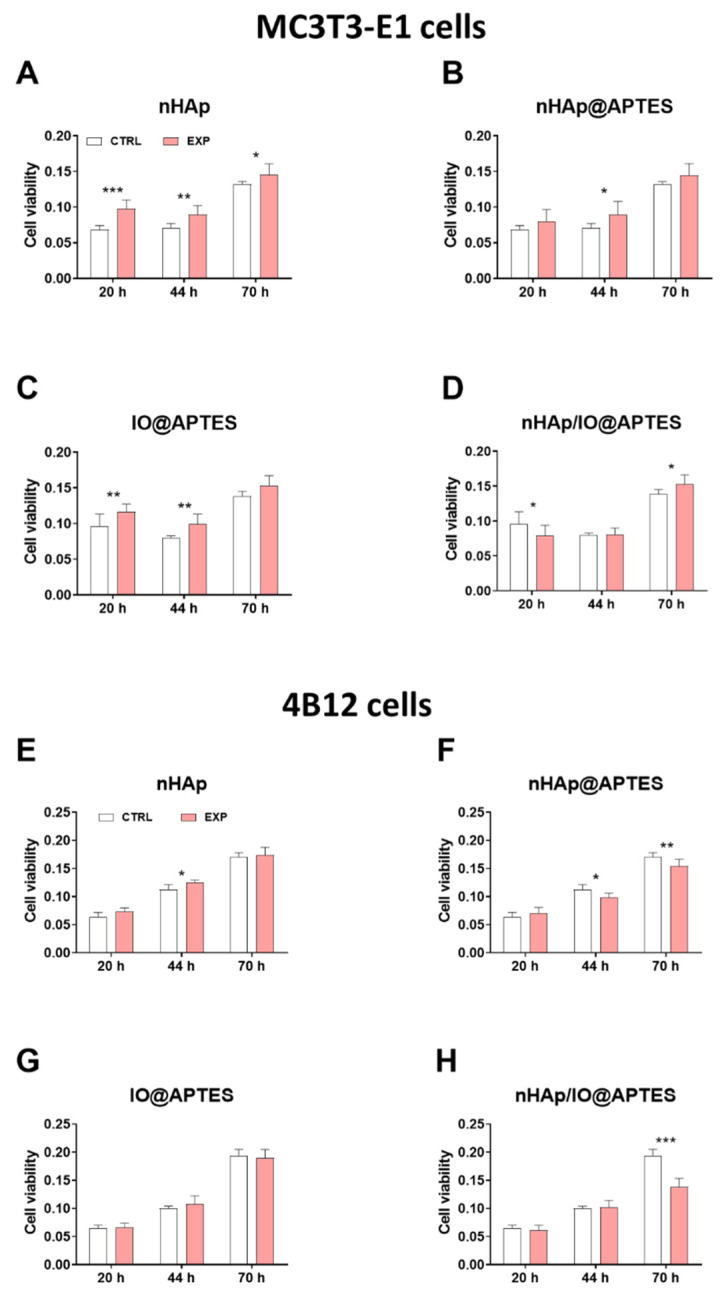

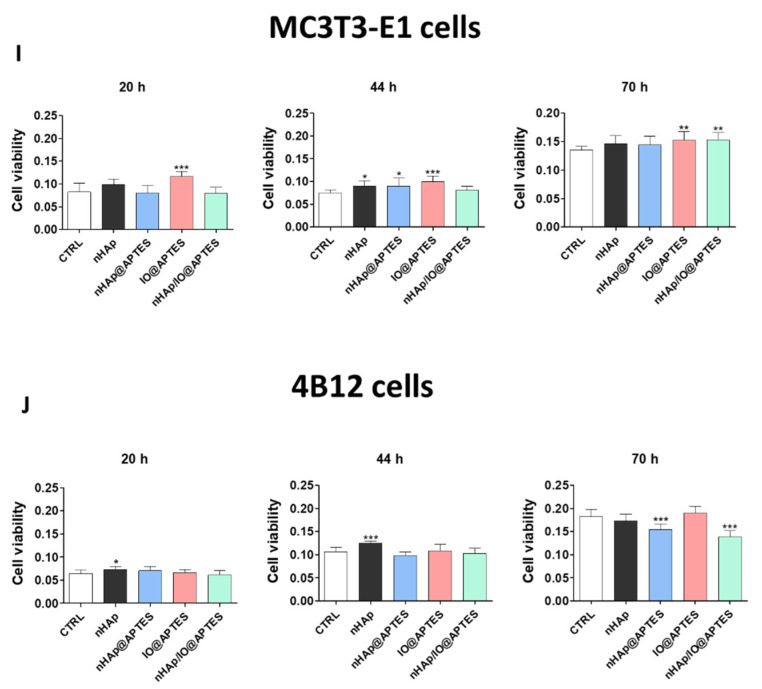
Evaluation of cytotoxicity. The impact of the nHAp (**A**), nHAp@APTES (**B**), IO@APTES (**C**), nHAp/IO@APTES (**D**) on the viability of the MC3T3-E1 cell line after 20, 44 and 70 h incubation and the nHAp (**E**), nHAp@APTES (**F**), IO@APTES (**G**), nHAp/IO@APTES (**H**) on the viability of the 4B12 cell line after 20, 44 and 70h incubation. Merged graphs for MC3T3-E1 (**I**) and 4B12 (**J**). The graphs represents mean values ± standard deviation. Significant differences are indicated as follows (* *p* < 0.05, ** *p* <0.01 and *** *p* < 0.001).

The kinetics changes of the viability of cells in the presence of biomaterials was determined after 20, 44 and 70 h of incubation on MC3T3-E1 cell line and 4B12 cell line (Figure 5A–D).

In relation to the control, we noticed the statistical significant increase of the cell viability on MC3T3-E1 cell line cultured in the presence of APTES modified biomaterials in each time point: after 20 h (Figure 5A), 44 h (Figure 5B,C) and after 70 h (Figure 5D). In turn, the new biomaterial (nHAp/IO@APTES) limited the cell viability after 20 h culturing (Figure 5D), while the initial biomaterial increasing it independently of time point (Figure 5A).

Similarly, we performed the analysis on the 4B12 cell line (Figure 5E–H) and we observed the statistical significant decrease of the viability after culturing in the presence of APTES modified biomaterials after 44 h and 70 h (nHAp@APTES—Figure 5F). The most expressive effect of the new synthesized biomaterial on the 4B12 cell line viability was seen after 70 h (Figure 5H). The impact of the new biomaterials on the mitochondria status was determined in relation to F-actin and MitoRed staining and the results were shown in Supplementary Figures S1 and S2. Obtained data was displayed on merged graphs in order to show cell viability for MC3T3-E1 (Figure 5I) and 4B12 (Figure 5J) at the same time.

### 3.5. Gene Expression Analysis

**Figure 6:**
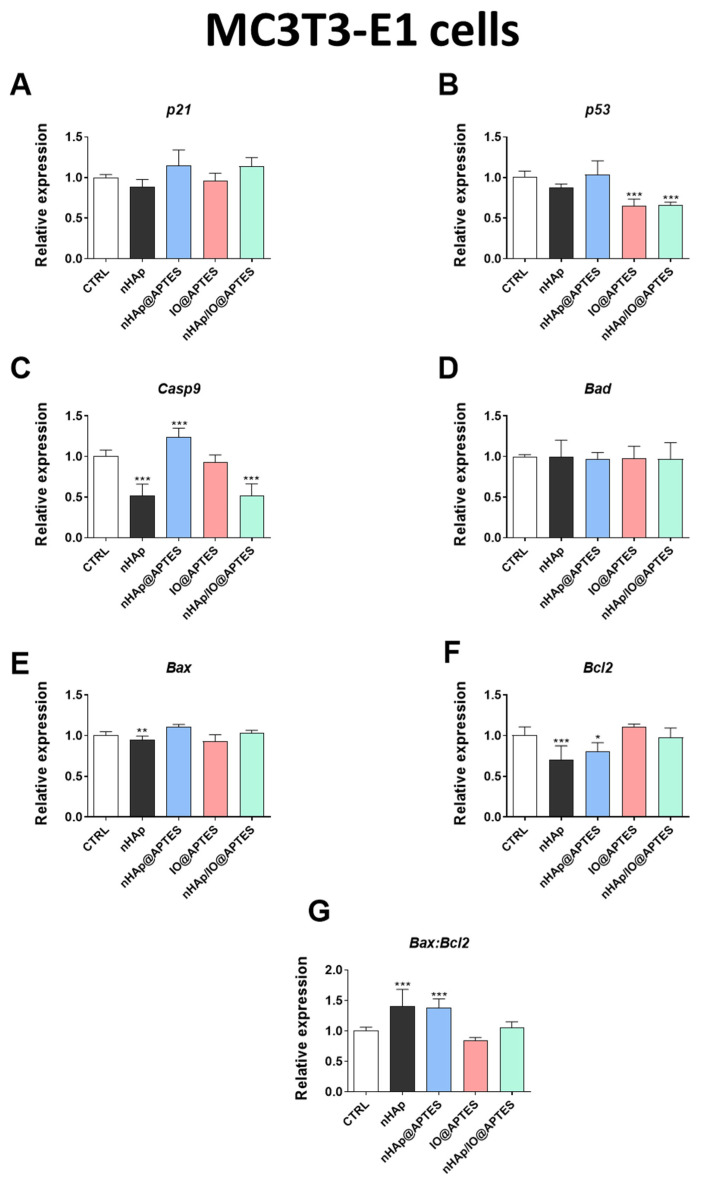

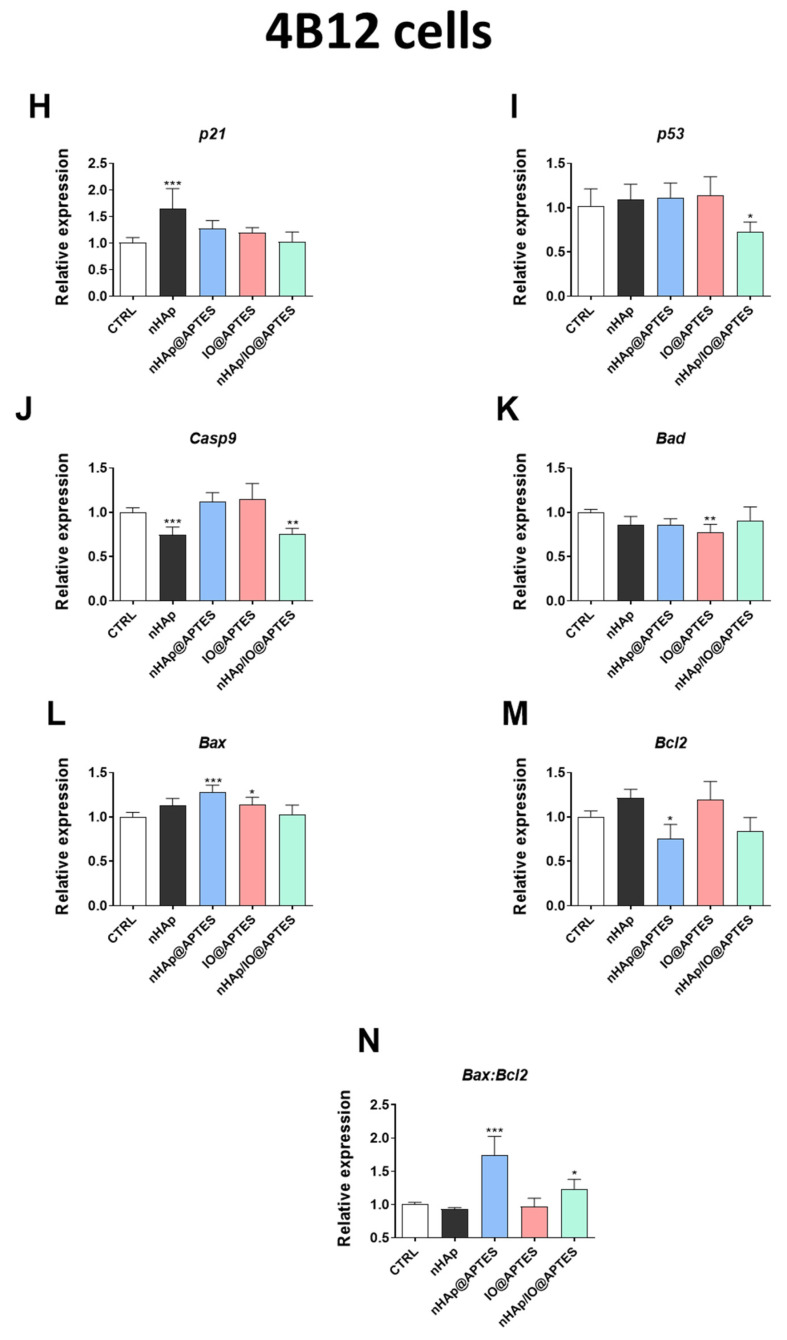
Evaluation of apoptosis. The impact of the biomaterials on the expression of genes associated with apoptosis: *p21* (**A**), *p53* (**B**), *Casp9* (**C**), *Bad* (**D**) *Bax* (**E**), *Bcl-2* (**F**) and *Bax/Bcl-2* ratio (**G**) after 20 h of incubation of MC3T3-E1 cell line. The impact of the biomaterials on the expression of genes associated with apoptosis: *p21* (**H**), *p53* (**I**), *Casp9* (**J**), *Bad* (**K**) *Bax* (**L**), *Bcl-2* (**M**) and *Bax/Bcl-2* ratio (**N**) after 20 h of incubation of MC3T3-E1 cell line. The graphs represent mean values ± standard deviation. Significant differences are indicated as follows: (* *p* < 0.05, ** *p* < 0.01 and *** *p* < 0.001).

Additionally, we performed real-time qPCR reactions to observe the impact of the new synthesized biomaterials on the expression of genes associated with apoptosis. Interestingly, none of the investigated materials affected p21 expression (Figure 6A). We noticed that the APTES-modified biomaterials significantly influenced the *p53* gene expression decrease (Figure 6B). Similarly, the new synthesized biomaterial (nHAp/IO@APTES) decreased the expression of *Casp9* (Figure 6C). The same effect of a decrease in *Casp9* gene expression was also observed in the case of the initial biomaterial in relation to the control, although the lack of IO in nHAp@APTES modification caused a strong increase in *Casp9* gene expression (Figure 6C). No differences were noted for Bad expression between groups (Figure 6D). We also observed a statistically significant decrease in the expression of *Bax* and *Bcl-2* after nHAp alone or nHAp@APTES stimulation (Figure 6E,F). Moreover, we observed an increase in the ratio of *Bax/Bcl-2* in the case of these two biomaterials (Figure 6G).

In the 4B12 cell line, none of the material affected expression of *p21* (Figure 6H). However, the new modified APTES biomaterial impacted the gene expression of *p53* and *Casp9* (Figure 6I,J) in the preosteoclastic cell line. In turn, the modification of the APTES biomaterial by adding Fe_3_O_4_ decreased the expression of *Bad* while concurrently increasing the expression of *Bax* (Figure 6K,L). The most evident effect on the expression of Bcl—family genes was exerted by nHAp@APTES. We observed that this biomaterial increased the expression of *Bax* (Figure 6L) and decreased that of *Bcl2* (Figure 6M) as well as the ratio of *Bax/Bcl2* (Figure 6N).

**Figure 7:**
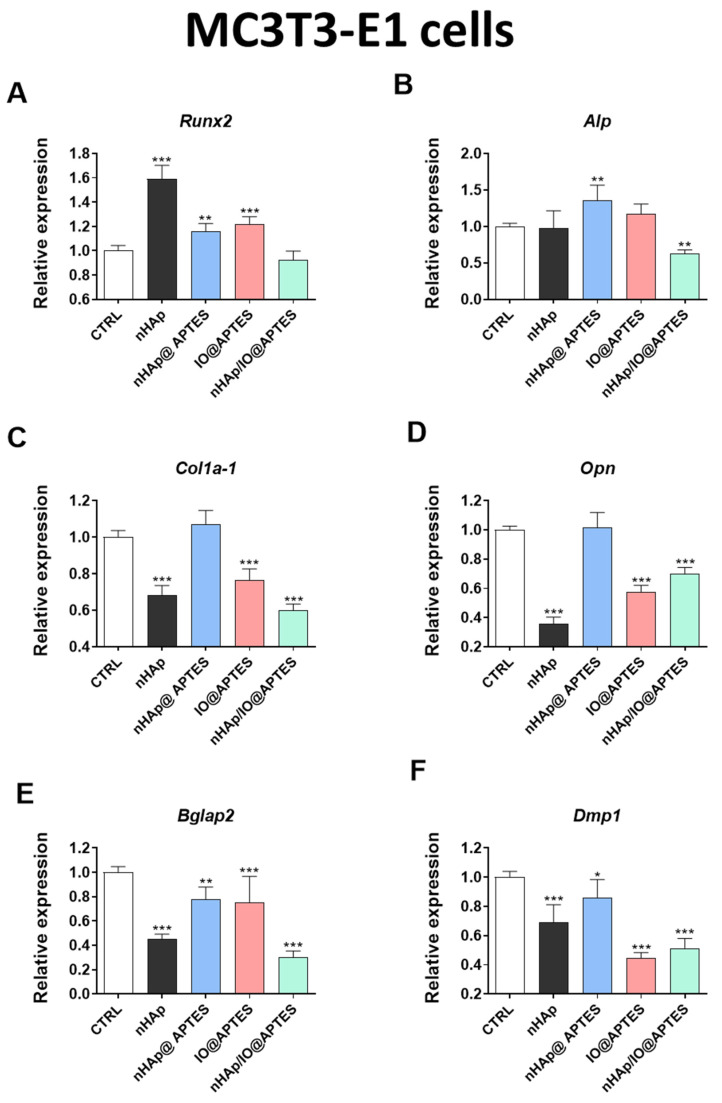

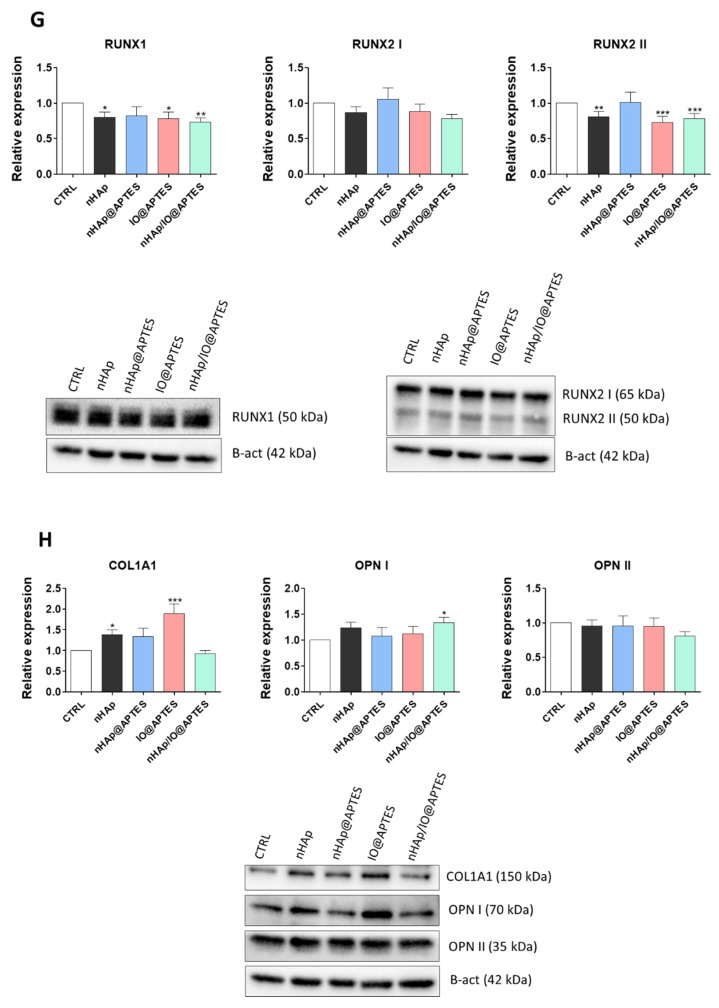

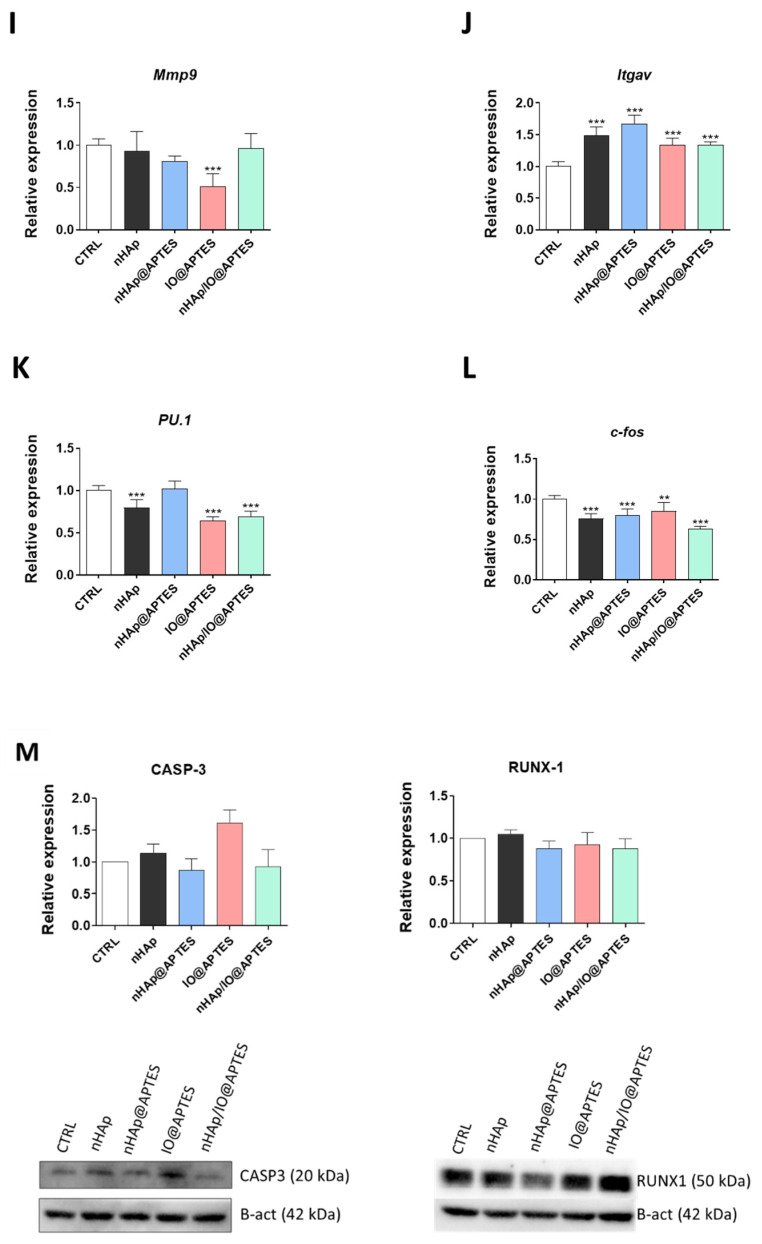
The influence of biomaterials on osteoblasts and osteoclasts. The impact of the biomaterials on the expression of genes associated with osteogenesis *Runx2* (**A**), *Alp* (**B**), *Col1a-1* (**C**), *Opn* (**D**), *Bglap2* (**E**) and *Dmp1* (**F**) after 20 h of incubation of MC3T3-E1 cell line and the results of Western blot for RUNX1, RUNX2 I, RUNX 2 II (**G**) and COL1A1, OPN I and OPN II (**H**). The impact of the biomaterials on the expression of genes associated with osteoclast activities *Mmp9* (**I**), *Itgav* (**J**), *PU.1* (**K**) and *c-fos* (**L**) after 20 h of incubation of 4B12 cell line. The amount of CASP-3 and RUNX-1 in osteoclasts (**M**). The graphs represent mean values ± standard deviation. Significant differences are indicated as follows: (* *p* < 0.05, ** *p* < 0.01 and *** *p* < 0.001).

To complete our knowledge about the impact of the biomaterials on the osteogenic and osteoclastogenic markers, we analyzed the gene expression typical of osteogenesis *Runx2* (Figure 7A), *Alp* (Figure 7B), *Col1a1* (Figure 7C), *Opn* (Figure 7D), *Bglap2* (Figure 7E) and *Dmp1* (Figure 7F). During the studies, we observed a statistically significant decrease in the majority of analyzed genes, including *Alp*, *Col1a-1*, *Opn*, *Bglap2* and *Dmp1*, after MC3T3-E1 cell line stimulation using the modified biomaterial (Figure 7B–F). Similar effects were observed in the case of IO@APTES stimulation, with the exception of *Alp* and *Runx-2*, which were observed to have opposite effects (Figure 7A–F). Additionally, the cells incubated in the presence of nHAp@APTES biomaterial showed a decrease in expression of *Runx2*, *Bglap2* and *Dmp1*, although in the case of *Alp*, the effect was changeable (Figure 7A–F). Moreover, nHAp alone exerted a significant decrease in the expression of *Col1A1*, *Opn*, *Bglap2* and *Dmp1* while increasing the expression of *Runx2* in this cell line (Figure 7A–F). Western blot analysis revealed that in comparison to the CTRL group, fabricated materials decreased levels of RUNX1 and RUNX2 II, while no differences were observed for the amount of RUNX2 I (Figure 7G). However, COL1A1 levels were increased in nHAp and IO@APTES groups and the OPN amount was increased in nHAp/IO@APTES, while no differences were observed for OPN II (Figure 7H).

Additionally, genes for osteoclast activity, including Mmp9 (Figure 7I), Itgav (Figure 7J), PU.1 (Figure 7K) and c-fos (Figure 7L), were investigated. The results of the gene expression linked with osteoclastogenic activities imply that the new biomaterial could influence the expression of Itgav (increase) and PU.1 or c-fos (decrease) in comparison to the control in the 4B12 cell line (Figure 7J,K). The other modifications of nHAp, e.g., nHAp@APTES, increased the expression of the Itgav and decreased the expression of c-fos (Figure 7J,L). The expression of Itgav was also increased after stimulation with IO@APTES, despite the fact that this biomaterial significantly decreased Mmp9 and c-fos expression. Western blot analysis revealed no statistically significant differences in the amount of CASP-3 or RUNX-1 in 4B12 cells (Figure 7M).

Additionally, we investigated expression of genes related to mitochondrial dynamics in both MC3T3-E1 and 4B12. The obtained results are presented in Supplementary Figures S3 and S4, respectively.

**Figure 8:**
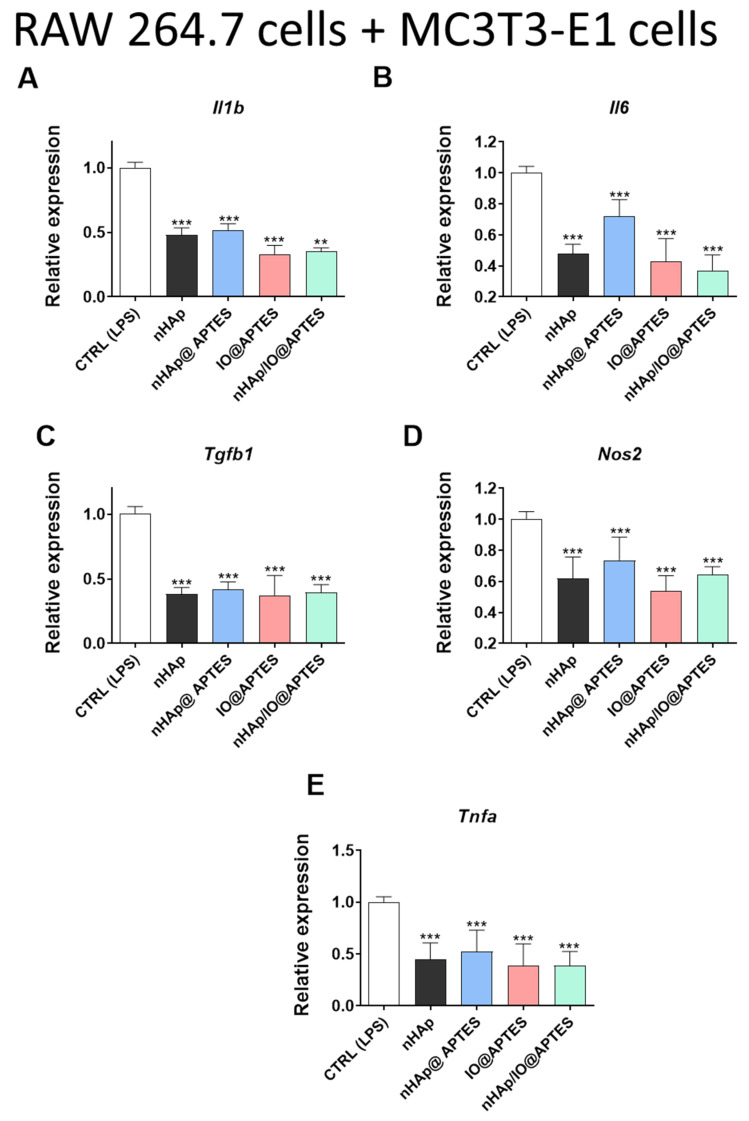

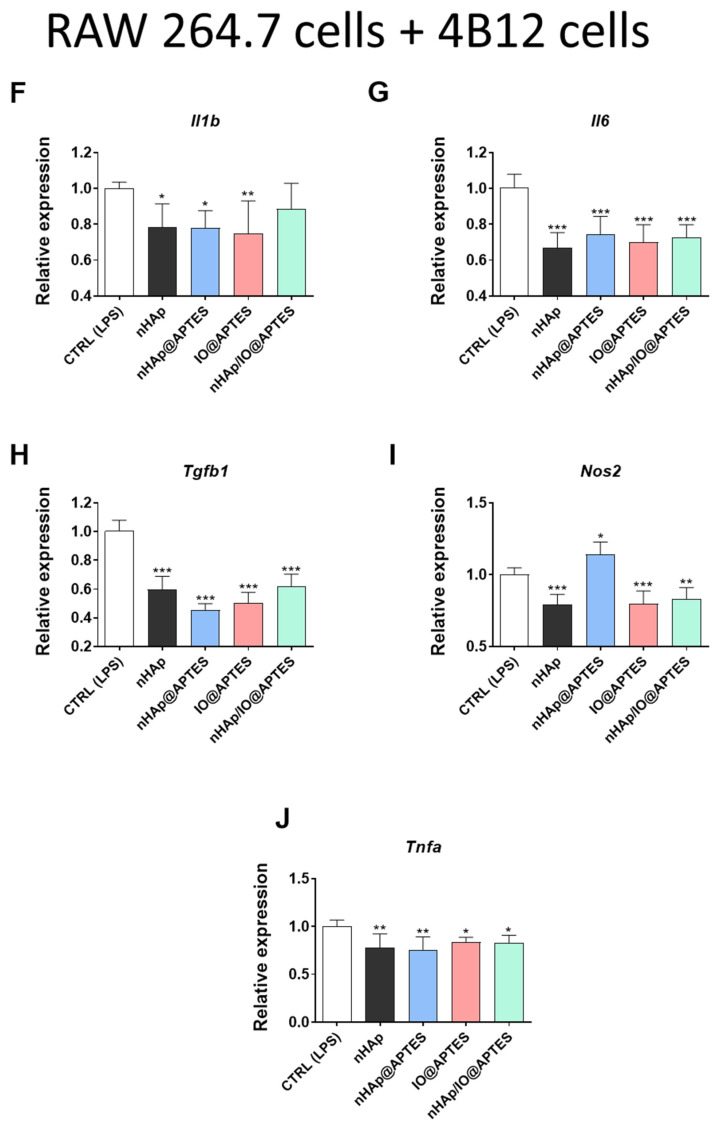
Assessment of inflammation. The impact of the biomaterials on the expression of genes associated with inflammation status *Il1b* (**A**), *Il6* (**B**), *Tgfb1* (**C**), *Nos2* (**D**) and *Tnfa* (**E**) after 20 h of incubation of MC3T3-E1 cell line. Expression of genes associated with inflammation status *Il1b* (**F**), *Il6* (**G**), *Tgfb1* (**H**), *Nos2* (**I**) and *Tnfa* (**J**) after 20 h of incubation of 4B12 cell line. The graphs represent mean values ± standard deviation. Significant differences are indicated as follows: (* *p* < 0.05, ** *p* < 0.01 and *** *p* < 0.001).

To evaluate the final impact of the new synthesized biomaterials on the inflammation state, we performed a coculture of RAW 264.7 and MC3T3-E1 and showed the changes in the proinflammatory cytokine profile. We observed a statistically significant decrease in the expression of *Il1b* (Figure 8A), *Il16* (Figure 8B), *Tgfb1* (Figure 8C), *Nos2* (Figure 8D) and *Tnfa* (Figure 8E) in almost all analyses using all kinds of biomaterial combinations (Figure 8A–E), except for an increase in *Nos2* expression after stimulation of IO@APTES (Figure 8D). Similar effects were observed in the 4B12 cell line after coculturing with RAW 264.7. All of the combinations of the new biomaterials decreased the expression of genes associated with the proinflammatory cytokines (Figure 8G–L).

## 4. Discussion

In recent years, osteoporosis (OP) prevalence has been rapidly increasing, and for that reason, represents a significant clinical challenge. Disease is usually treated with anabolic (e.g., parathyroid hormone) and antiresorptive (e.g., estrogen, bisphosphonates) agents which stimulate or inhibit bone formation, respectively. Despite therapy, as a result of decreased bone mass, elderly patients are prone to life-threatening fractures. As their occurrence in the USA is estimated at one and half million each year, fractures challenge the medical care system and economy. Over two million surgeries are conducted each year to repair damaged or fractured bones by grafting [51], and as a consequence, the biomaterial market exceeded USD 39 billion in 2013 [52]. What is more, due to abnormalities in bone homeostasis, the healing process is strongly limited and the failure rate of implant fixation is relatively high (more than 50%) [53]. For that reason, there is an urgent need to develop novel biomaterials, fixation plates and screws designed to meet the needs of OP patients and effectively stimulate the regeneration process.

One approach to fabricating that kind of personalized scaffold is targeting the disbalance between osteoblast and osteoclast activity, which is a major contributor to the decreased healing capacity of OP-affected bone. Yet, nowadays, most of the materials still focus heavily on osteoinductive and mechanical properties rather than on molecular mechanisms, which leads to a limited healing capacity. In the presented study, we decided to compare the materials which are characterized by great osteoinductive and mechanical properties and select the most potent of them in the context of their bioactivity, interpreted as the ability to modulate bone cells’ fate. Bone tissue constitutes both organic components—mostly collagen fibers—and inorganic components, including calcium (Ca) and phosphorus (P) in the form of hydroxyapatite (nHAp) crystals. For that reason, nHAp has been widely investigated as a compound for multiple polymers, growth factors and stem-cell-based therapies, which proved its osteoinductivity properties and potency in bone healing [15,16,54,55,56]. More recently, special attention has been paid to magnetic nanoparticles (MNPs), which can be integrated into the wide spectrum of polymers and enhance their bioactivity. What is more, due to ferromagnetic nature of MNPs, they can be used to fabricate a novel class of so-called smart biomaterials whose properties can be controlled and modulated by the application of external factors, including, in this scenario, a magnetic field. Typical and well-studied MNPs for bone regeneration include Fe_3_O_4_ (IO) characterized by good bioactivity and cytocompatibility. It was shown that composites, including Fe_3_O_4_, stimulate bone regeneration and enhance activity of mesenchymal stem cells (MSC) [57,58]. Yet, besides these two well-known proteogenic factors, in the presented study we decided to introduce a compound which has never been studied before in the context of bone regeneration and compare its properties with counterparts well-described in the literature. APTES has been previously applied to silica-based nanomaterials in order to allow grafting of selected molecules. Yet, little is known regarding APTES in combination with pro-osteogenic factors, including nHAp and Fe_3_O_4_. This is especially important as pure APTES molecules possess different properties than their nanoassembled forms. What is more, APTES has been used to immobilize cells to bind them to the surface of a glass slide [59]. Herein, we investigated how nHAp, nHAp@APTES, IO@APTES and nHAp/IO@APTES composites affected the metabolic properties of preosteoblasts (MC3T-E1 cell line) and preosteoclasts (4B12 cell line) with special attention to cytotoxicity, apoptosis, mitochondrial dynamics, inflammation and bone formation/resorption properties, which are all crucial in the healing of OP-affected bone.

Incorporation of magnetic IO nanoparticles into the nHAp structure may result in the formation of a material responsive to an external magnetic field, which is highly desirable for drug delivery systems or hyperthermia [60,61], whereas the additional modification with the APTES polymer layer leads to higher biocompatibility through biofunctionalization of amine groups [62,63]. Several attempts have been made to combine nHAp with IO [21,25,64], as well as covering both materials with a polymer layer [8,10,36,38,65,66]. In this research, we focused on covering and functionalizing pure nHAp, IO and nHAp/IO composites with APTES. All desired materials were successfully obtained at the nanometric scale, and the composite material exhibited superparamagnetical and ferrimagnetical behavior at 300 K and 2 K, respectively. Moreover, the material had a low magnetization value due to its nanometer scale and diamagnetic contribution of nHAp in the composite. The research shows, using infrared measurements and electron microscopy, that wet chemistry synthesis followed by coating with a polymer layer on the surface was an effective method for obtaining highly functional nanoparticles. However, the TEM images show that nHAp/IO@APTES generated a highly agglomerated material composed of 72% and 28% of nHAp and IO, respectively. The other studies devoted to manufacturing the nHAp/IO composite found in the literature showed a similar tendency towards mutual agglomeration of the particles [67,68]. However, a core–shell system with IO as a core and an nHAp shell was found as well [21,69]. Comparison between the studies showed that the nHAp-to-IO ratio can be considered as a factor determining the form of a composite. A higher amount of nHAp precursors leads to fabrication of the individual particles, which further agglomerates with the IO nanoparticles. In contrast, a lower nHAp content (a maximum of 20 mol% Ca^2+^: IO) resulted in its crystallization on the IO surface, forming an overlaying cover [69]. Herein, the nHAp and IO were mixed in a 2:1 mass ratio, leading to generation of two individual phases connected by the physical agglomeration.

Biocompatibility of all tested conjugates was shown with resazurin-based assays for both MCT3-E1 and 4B12 cell lines, and no cytotoxicity was noted. Interestingly, nHAp and IO@APTES were found to enhance cellular proliferation. Multiple lines of evidence confirm that nHAp and IO are able to enhance cellular growth; yet, for APTES, existing data are contradictory. Our own studies have shown that modification of poly (L-lactic acid) with nHAp improved functionality of human-adipose-derived stromal cells (hASCs) through increased viability and enhanced mitochondrial activity [64]. What is more, biphasic polyurethane–polylactide sponges doped with nHAp enhanced hASC proliferation and chondrogenic differentiation [65]. On the other hand, APTES immobilized onto the silica nanoparticle surfaces inhibited growth of human endothelial cells, which has not been found in either free APTES or unmodified silica nanoparticles [66]. When taking into consideration the application of scaffolds in OP fracture healing, it is not only important to promote osteoblast proliferation but also to diminish the activity of osteoclasts, which causes progressive bone resorption and leads to decreased bone mass. Interestingly, here we have found that none of the tested materials enhanced osteoclast proliferation and two among them—nHAp@APTES and nHAp/IO@APTES—significantly reduced growth kinetics of 4B12, which further supports APTES application in scaffolds for bone regeneration.

To support data from cytotoxicity assays and confirm the safety of APTES in biomedical application, we performed RT-qPCR to investigate the expression of apoptosis-related genes after 20 h of culturing with tested compounds. Interestingly, no differences in the expression in p21 nor Bad were noted between investigated groups, whereas nHAp@APTES and nHAp/IO@APTES significantly downregulated the expression of p53 in comparison to the CTRL group. What is more, these two combinations had no effect on the Bax-to-Bcl2 ratio, which further confirms their biocompatibility and cytoprotective effects in MC3T3-E1 cells. Osteoblast apoptosis contributes to OP progression, and targeting the pathway in the early stage of the disease may be beneficial [70]. Next, it was investigated whether tested chemicals modulate the apoptosis of 4B12 cells. nHAp/IO@APTES was most potent in the induction of that process in the osteoclast while at the same time promoting the osteoblasts’ growth, which strongly supports its application in OP. Interestingly, it is the first report showing bidirectional modulation of cell fate by APTES.

In accordance with recent data which revealed the profound role of mitochondria in osteogenesis, osteoclast activity and bone loss, we investigated how tested substances affect expression of genes involved in the regulation of mitochondria dynamics [71]. In vitro assays demonstrated that the most profound effects on mitochondria in MC3T3-E1 were exerted by nHAp@APTES, which stimulated not only fission and fusion but also mitochondrial removal. Interestingly, other materials decreased expression of all investigated genes. Sun et al. [72] found that mitophagy was triggered in MC3T3-E1 cells by 17β-estradiol and increased cell proliferation, highlighting the significance of that hormone for the clinical treatment of OP. Pink1-induced mitophagy is also crucial for preosteoblast differentiation. These findings indicate that nHAp@APTES promotes mitochondrial biogenesis and their function. On the other hand, the same chemicals decreased expression of Pink1 in 4B12 cells, which indicated mitophagy amelioration.

Next, we investigated the expression of genes which regulate osteoblast and osteoclast functions in cells treated with all tested materials. For MC3T3-E1, we found that nHAp@APTES enhanced the expression of Runx2, Alp and Col1a-1, which supports its proosteogenic role. Interestingly, other compounds decreased expression of marker genes, except Runx2, whose mRNA levels were significantly enhanced in the nHAp group. Yet, it should be taken into consideration that Runx2 is an early marker of osteogenic differentiation. It was previously shown by our group that Fe_3_O_4_ nanoparticles can be applied for the fabrication of biomaterials for OP patients as they improve osteogenesis via the Runx2 pathway and decrease osteoclastogenesis by triggering apoptosis; however, in the combination with APTES, such effects were limited [27]. In the case of osteoclasts, materials doped with APTES decreased expression of Mmp9, PU.1 and c-fos; however, when taking into consideration data from apoptosis analysis, nHAp@APTES most significantly inhibited osteoclast activity among tested compounds.

Inflammation is one of the key feature of OP, and Il-1b can induce experimental osteoarthritis in chondrocytes [73]. Multiple cytokines were shown to accelerate bone resorption, including Il-, Tnf, Il-11, Il-6, Il-15 and Il-17 [74]. To investigate the crosstalk between osteoclasts and osteoblasts with immune cells, we performed a coculture with RAW264.7 macrophages and investigated how it affects cytokine expression profile. We noted a significant decrease in Il-1b expression among all investigated groups in each cell type. Cytokine is a strong stimulator of in vitro and in vivo bone resorption; thus, its inhibition may contribute to enhancing bone regeneration and healing in OP patients [74]. Similar, the Il-8 levels were increased in OP patients as well [75]. Obtained data support application of materials incorporated with APTES for the treatment of OP-affected bone due to their anti-inflammatory properties.

## 5. Conclusions

To summarize, in the presented study we tested different type of materials incorporated with APTES in order to verify their cytocompatibility and ability to modulate bone cells’ metabolism. We have shown that APTES can be applied in the fabrication of personalized biomaterials for the treatment of osteoporotic bone fractures as it is nontoxic and enhances the metabolic activity of preosteoblasts while diminishing activity of osteoclasts. Furthermore, it does not elicit an immune response and decreases expression of selected proinflammatory cytokines. APTES materials affect the key features of OP, which supports their application in the fabrication of novel biomaterials.

## Figures and Tables

**Table 1:** List of antibodies used in western blot.

Protein	Dilution	Manufacturer, Catalog No.
CASP3	1:500	Sigma Aldrich, C8487
COL1A1	1:50	Sigma Aldrich, A2066
RUNX1	1:200	Santa Cruz, sc-365644
RUNX2	1:100	Santa Cruz, sc-390351
OPN	1:1000	Abcam, ab8448
Β-ACT	1:2500	Sigma Aldrich, A5441

**Table 2:** List of primers used in RT-qPCR. Alp—alkaline phosphatase, Bad—BCL2-associated agonist of cell death, Bax—bcl-2-like protein 4, Bcl2—B-cell lymphoma 2, Bglap2—osteocalcin-2, Casp9—caspase 9, c-fos—proto-oncogene c-fos, Colla-1—alpha-1 type I collagen, Dmp1—dentin matrix acidic phosphoprotein 1, Gapdh—glyceraldehyde 3-phosphate dehydrogenase, Il-1b—interleukin 1 beta, Il6—interleukin 6, Itgav—integrin subunit alpha V, Mff—mitochondrial fission factor, Mfn1—mitofusin 1, Mmp9—matrix metallopeptidase 9, Nos2—nitric oxide synthase 2, Opn—osteopontin, p21—cyclin-dependent kinase inhibitor 1, p53—tumor suppressor p53, Pink1—PTEN-induced kinase 1, PU.1—transcription factor PU.1, Runx2—runt-related transcription factor 2, Tgfb1—transforming growth factor beta 1, Tnfa—tumor necrosis factor alpha.

Gene	Primers (5′→3′)	Length of Amplicon	Accession No.
*Alp*	F: TTCATAAGCAGGCGGGGGAG R: TGAGATTCGTCCCTCGCTGG	198	NM_007431.3
*Bad*	F: ACATTCATCAGCAGGGACGG R: ATCCCTTCATCCTCCTCGGT	115	NM_001285453.1
*Bax*	F: AGGACGCATCCACCAAGAAGC R: GGTTCTGATCAGCTCGGGCA	251	NM_007527.3
*Bcl2*	F: GGATCCAGGATAACGGAGGC R: ATGCACCCAGAGTGATGCAG	141	NM_009741.5
*Bglap2*	F: CTCCTGAGAGTCTGACAAAGCCTT R: GCTGTGACATCCATTACTTGC	100	NM_001032298.3
*Casp9*	F: CCGGTGGACATTGGTTCTGG R: GCCATCTCCATCAAAGCCGT	278	NM_001355176.1
*c-fos*	F: CCAGTCAAGAGCATCAGCAA R: TAAGTAGTGCAGCCCGGAGT	248	NM_010234.3
*Col1a-1*	F: CCAGCCGCAAAGAGTCTACA R: CAGGTTTCCACGTCTCACCA	175	NM_007742.4
*Dmp1*	F: CCCAGAGGCACAGGCAAATA R: TCCTCCCCAATGTCCTTCTT	211	NM_001359013.1
*Gapdh*	F: TGCACCACCAACTGCTTAG R: GGATGCAGGGATGATGTTC	177	NM_001289726.1
*Il-1b*	F: TGCCACCTTTTGACAGTGATG R: TGATGTGCTGCTGCGAGATT	138	NM_008361.4
*Il6*	F: GAGGATACCACTCCCAACAGACC R: AAGTGCATCATCGTTGTTCATACA	141	NM_001314054.1
*Itgav*	F: ACAATGTAAGCCCAGTTGTGTCT R: TTTGTAAGGCCACTGGAGATTTA	236	NM_008402.3
*Mff*	F: TCACATTTGGTGAGTGGGGC R: TTTTCCGGGACCCTCATTCG	125	NM_001372412.1
*Mfn1*	F: ATCACTGCAATCTTCGGCCA R: AGCAGTTGGTTGTGTGACCA	283	NM_024200.4
*Mmp9*	F: TTGCCCCTACTGGAAGGTATTAT R: GAGAATCTCTGAGCAATCCTTGA	172	NM_013599.4
*Nos2*	F: GACAAGCTGCATGTGACATC R: GCTGGTAGGTTCCTGTTGTT	325	NM_001313922.1
*Opn*	F: AGACCATGCAGAGAGCGAG R: GCCCTTTCCGTTGTTGTCCT	340	NM_001204203.1
*p21*	F: TGTTCCACACAGGAGCAAAG R: AACACGCTCCCAGACGTAGT	175	NM_001111099.2
*p53*	F: AGTCACAGCACATGACGGAGG R: GGAGTCTTCCAGTGTGATGATGG	287	NM_001127233.1
*Pink1*	F: CCCCAGTGCGGTAATTGACT R: CTAGAAGATGCTCGCCCCAG	281	NM_026880.2
*PU.1*	F: GAGAAGCTGATGGCTTGGAG R: TTGTGCTTGGACGAGAACTG	175	NM_001378899.1
*Runx2*	F: TCCGAAATGCCTCTGCTGTT R: GCCACTTGGGGAGGATTTGT	130	NM_001271630.1
*Tgfb1*	F: GGAGAGCCCTGGATACCAAC R: CAACCCAGGTCCTTCCTAAA	94	NM_011577.2
*Tnfa*	F: ACAGAAAGCATGATCCGCGA R: CTTGGTGGTTTGCTACGACG	295	NM_013693.3

## Data Availability

The datasets generated during and/or analyzed during the current study areavailable from the corresponding author on reasonable request.

## Supplementary Materials

The following are available online at https://www.mdpi.com/article/10.3390/ma15062095/s1, Figure S1. The impact of the control (untreated) (A), nHAp (B), nHAp@APTES (C), Fe_3_O_4_ +APTES (D) and nHAP + Fe_3_O_4_ +APTES (E) on the mitochondria status of MC3T3-E1 cell line after 20 h of incubation, Figure S2. The impact of the control (untreated) (A), nHAP (B), nHAp@APTES (C), IO@APTES (D) and nHAp/IO@APTES (E) on the mitochondria status of 4B12 cell line after 20 h of incubation, Figure S3. The impact of the biomaterials on the expression of genes associated with mitochondrial dynamics Mff (A), Mfn1 (B), Pink1 (C) after 20h incubation of MC3T3-E1 cell line. Figure S4. The impact of the biomaterials on the expression of genes associated with mitochondrial dynamics Mff (A), Mfn1 (B), Pink1 (C) after 20h incubation of 4B12 cell line.
