# Supplementary material for: Aminopropyltriethoxysilane (APTES)-Modified Nanohydroxyapatite (nHAp) Incorporated with Iron Oxide (IO) Nanoparticles Promotes Early Osteogenesis, Reduces Inflammation and Inhibits Osteoclast Activity
Source: Materials (Basel). 2022 Mar 11;15(6):2095. doi: 10.3390/ma15062095 (PMC8953252; doi:10.3390/ma15062095)
Supplement: Supplementary file 1 [file materials-15-02095-s001.zip › materials-1572800-supplementary.pdf]

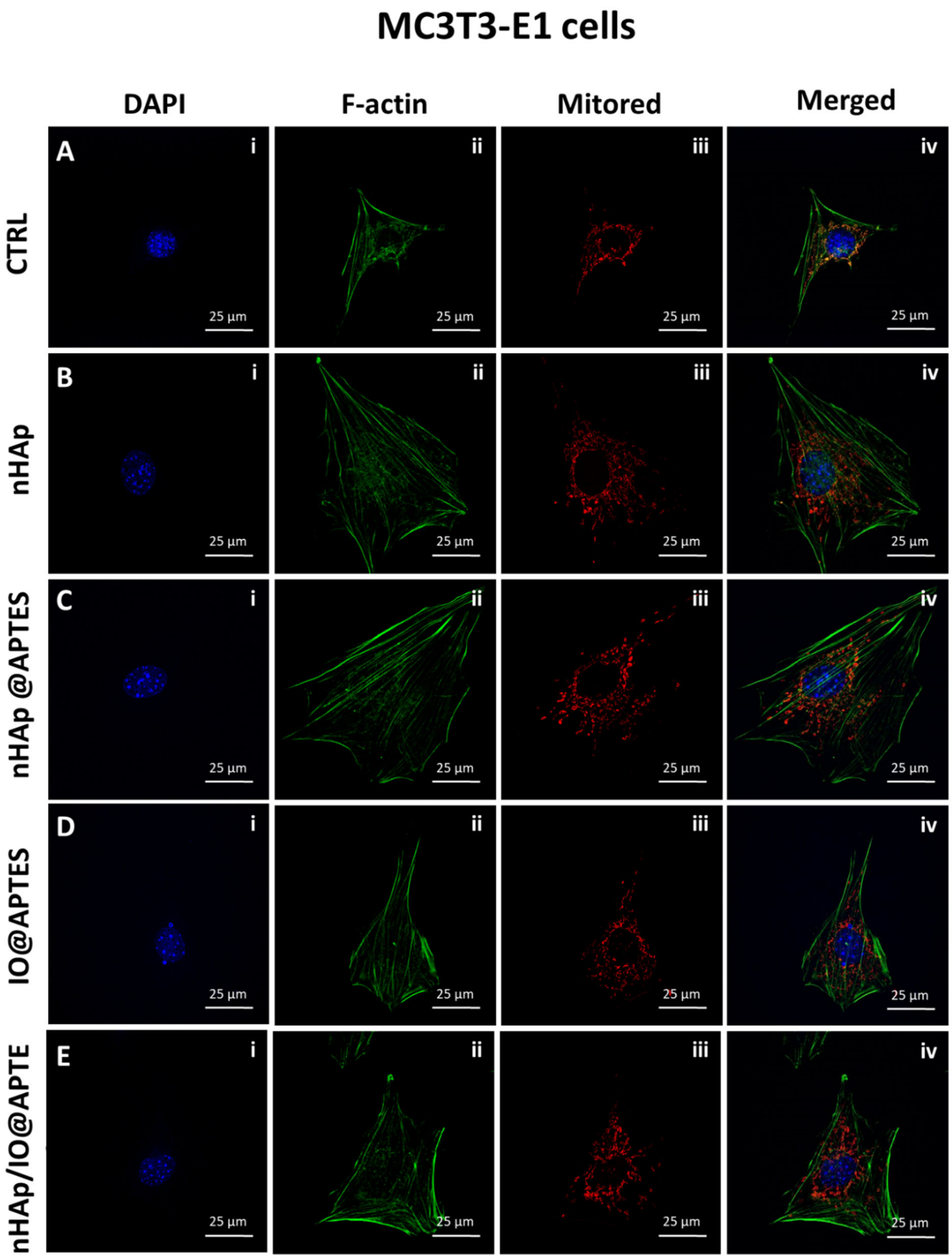

Figure S1. The impact of the control (untreated) (**A**), nHAp (**B**), nHAp@APTES (**C**), Fe3O4 +APTES (**D**), nHAP + Fe3O4 +APTES (**E**) on the mitochondria status of MC3T3-E1 cell line after 20h of incubation. The photographs were captured on the magnification 600×.

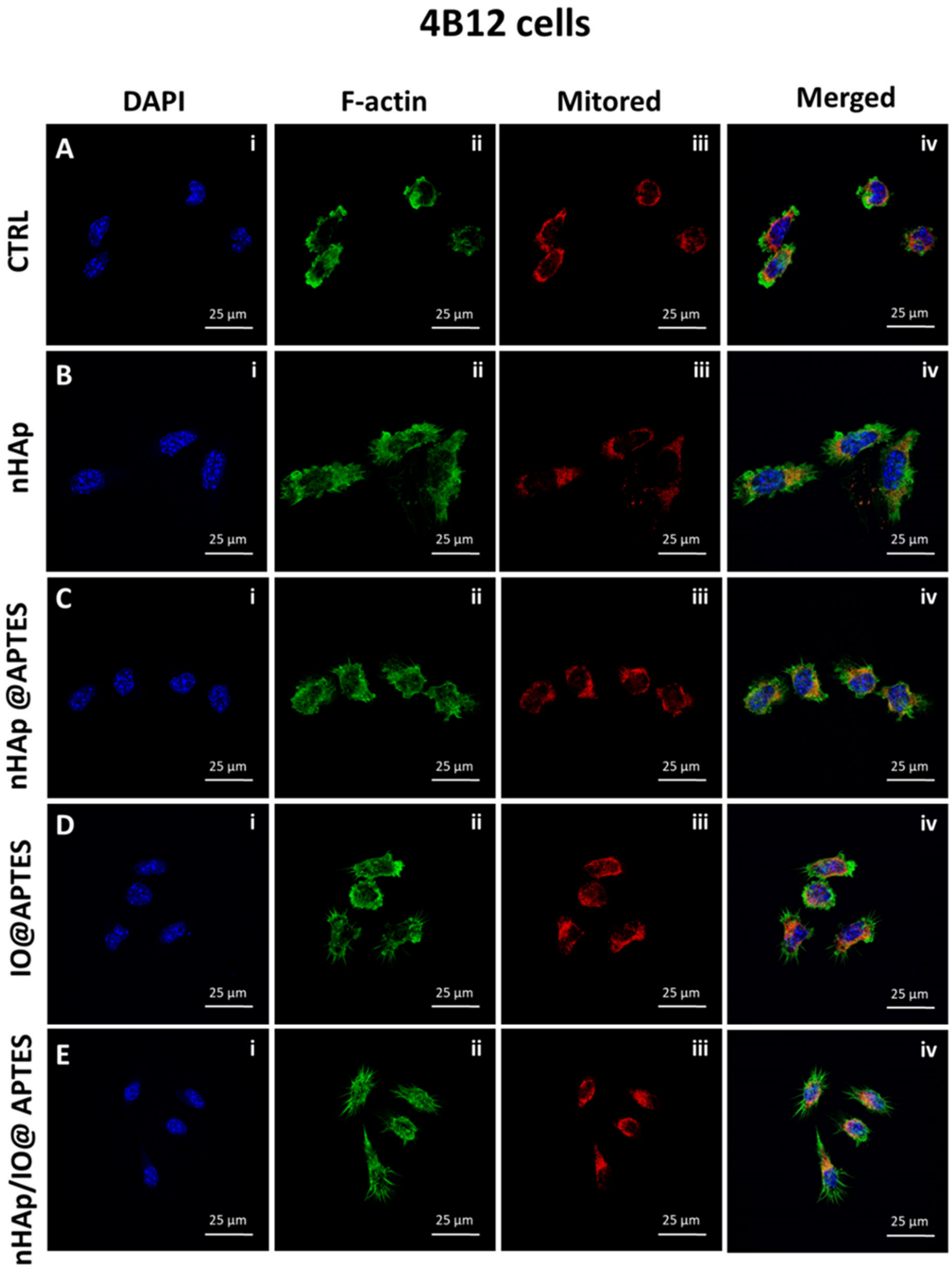

Figure S2. The impact of the control (untreated) (**A**), nHAP (**B**), nHAp@APTES (**C**), IO@APTES (**D**), nHAp/IO@APTES (**E**) on the mitochondria status of 4B12 cell line after 20h of incubation. The photographs were captured on the magnification 600x.

In case of 4B12 cell line, we have found that mitochondrial activity in comparison to control group (Figure S1A) is diminished in nHAP (Figure S1B) and nHAp@APTES (Figure S1C). Remaining groups (Figure S1D and Figure S1E) displayed similar mitochondria fluorescence intensity to control group.

In case of 4B12 cell line, we have found that mitochondrial activity in comparison to control group (Figure S2A) is diminished in nHAP (Figure S2B) and nHAp@APTES (Figure S2C). Remaining groups (Figure S2D and Figure S2E) displayed similar mitochondria fluorescence intensity to control group.

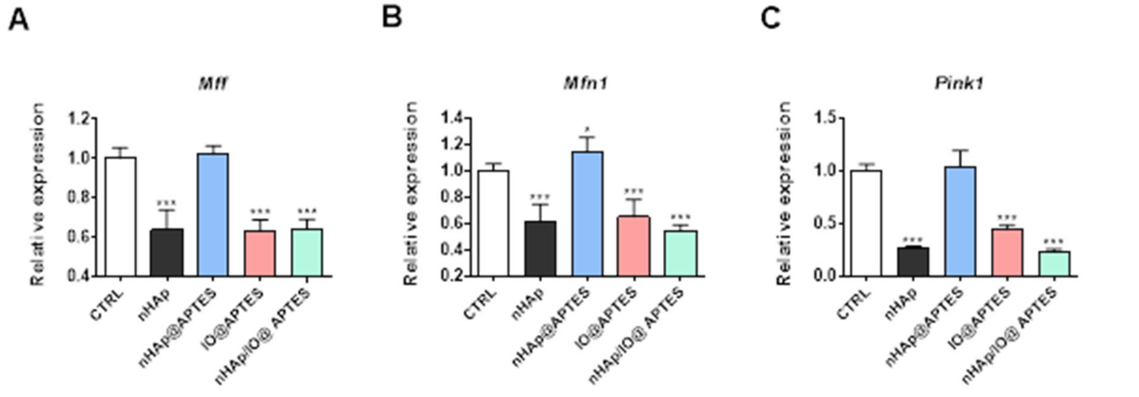

Figure S3. The impact of the biomaterials on the expression of genes associated with mitochondrial dynamics Mff (**A**), Mfn1 (**B**), Pink1 (**C**) after 20h incubation of MC3T3-E1 cell line. The graphs represent mean values ± standard deviation. Significant differences are indicated as follows (* $p < 0,05$, ** $p < 0,01$ and *** $p < 0,001$)

In order to complete information about the impact of the biomaterials on the mitochondrial dynamics, we assessed the gene expression of the Mff, Mfn1 and Pink1 on the MC3T3-E1 and 4B12 cell line after 20h of incubation.

Modified new biomaterial nHAp/IO@APTES can influence on the genes involved in the mitochondrial dynamics, we observed the statistically significant decrease of all three genes. Similar effect was also observed in case of nHAP and its modification by adding Fe3O4. Only the Mfn1 gene expression after APTES modified nHAP was increase on the MC3T3-E1 cell line (Figure S3A, Figure S3B and Figure S3C).

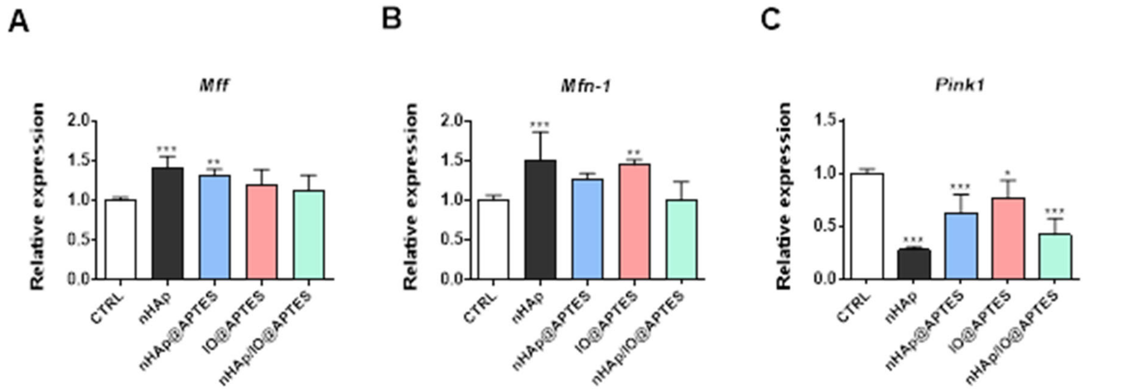

Figure S4. The impact of the biomaterials on the expression of genes associated with mitochondrial dynamics Mff (**A**), Mfn1 (**B**), Pink1 (**C**) after 20h incubation of 4B12 cell line. The graphs represent mean values ± standard deviation. Significant differences are indicated as follows (* p < 0,05, ** p < 0,01 and *** p < 0,001).

In 4B12 cell line, enhanced expression of Mff was observed in nHAp (Figure S4A) and nHAp@APTES (Figure S4B) groups. In turn, on 4B12 cell line the effect of the tested new biomaterial was observed only in case of Pink1 gene, where the expression was significantly decrease in relation to control (Figure S4C). Meanwhile the effect of the IO @APTES was quite different and according to the Mfn1 it was decrease, while according to the Pink1 it was increase (Figure S4B and Figure S4C). Similar effects were observed during the studies using nHAP@APTES, the Mff expression was increased but the Pink-1 expression is decreased on 4B12 cell line (Figure S4A and S4C). Taking into the consideration the obtained results we noticed that the strongest effect was associated with the initial biomaterial – nHAp, which caused the increase of Mff and Mfn-1 gene expression, at the same time caused the decrease of Pink-1 gene expression (Figure S4A–S4C).
